# Public health approaches to gambling: a global review of legislative trends

**DOI:** 10.1016/S2468-2667(23)00221-9

**Published:** 2023-11-07

**Authors:** Daria Ukhova, Virve Marionneau, Janne Nikkinen, Heather Wardle

**Affiliations:** a School of Social and Political Sciences, University of Glasgow, Glasgow, UK; b Centre for Research on Addiction, Control and Governance, University of Helsinki, Helsinki, Finland

## Abstract

The public health community has called for governments to recognise the harms associated with gambling, and for gambling policies to include population-based harm prevention approaches. This Health Policy explores the translation of this call into global policy action by systematically reviewing legislation of jurisdictions that introduced major gambling legislation change (ie, restricting or extending gambling provision) between Jan 1, 2018, and Dec 31, 2021. We mapped the global availability of legal gambling and changes in its provision, and conducted critical frame analysis on a sample of 33 jurisdictions introducing major policy change to assess the extent to which the protection of health and wellbeing was embedded within legislation. More than 80% of countries worldwide now legally permit gambling. Harmful gambling was recognised as a health and wellbeing issue in most of the analysed jurisdictions, but near-exclusive focus was given to individual-level harms rather than to wider social and economic harms, or harms to others. Most of the proposed prevention measures focused on individual responsibility. Gambling policies worldwide are changing, but addressing gambling as a public health issue is not yet translating into comprehensive policy action across jurisdictions.

## Introduction

The concept of gambling as a public health issue has a long history. In 1994, Volberg argued that gambling should be viewed through a public health lens.^1^ In 1999, Korn and Shaffer^2^ further contended that a whole-system approach was needed to prevent harms, with a focus on individual action but also on the structures of gambling provision. Over the past 5 years, researchers have called for a broader public health-based approach to gambling harms.^3^, ^4^, ^5^, ^6^, ^7^ However, systematic assessment of whether these calls have been translated into policy making has not been done.

Gambling policy debate tries to reconcile different approaches to the prevention of harms. Part of this debate focuses on whether prevention efforts should be primarily targeted at so-called vulnerable individuals, such as people experiencing gambling disorders, or rather at the systemic, whole-population level, while also recognising that a comprehensive public health prevention strategy would include both.^8^, ^9^, ^10^ Scholars have noted a preference among many policy makers for targeted prevention activity, with responsibility for action focused on individuals.^5^, ^11^, ^12^ This debate reflects other public policy areas (eg, obesity and health-care financing) in which Chater and Loewenstein have highlighted the ongoing competition between system-frame and individual-frame perspectives on societal challenges and their solutions.^13^

Extending critical perspectives from sociology and public health,^14^, ^15^, ^16^, ^17^ Chater and Loewenstein define individual-frame perspectives as those that focus on individual frailties and vulnerabilities that are deemed responsible for the harms they engender. Individual-frame interventions “don't fundamentally change the rules of the game, but make subtle adjustments to help fallible individuals play the game better”.^13^ When applied to gambling, individual-frame policies and interventions highlight individual responsibility and self-regulation^18^, ^19^, ^20^ and include self-management tools, responsible gambling awareness campaigns, education about gambling harms, feedback on personal patterns of consumption, and behavioural algorithms with player data to identify people at harm.^9^, ^21^

These policies and interventions are now standard features of many corporations' so-called responsible gambling or safer gambling strategies and are frequently used as the first line of prevention activity.^22^ However, the primacy of individual-frame approaches often means that broader, structural, and system-wide initiatives are sidelined.^13^ System-frame approaches focus on the systems, rules, and norms governing our institutions. Their application to gambling includes (among others): the regulation of products, including their design and characteristics; the nature and extent of gambling advertising and marketing; the accessibility, availability, and geolocation of gambling products and premises; and the level, form, and nature of taxation applied to the product.^9^, ^21^

The dominance of individual-frame perspectives in gambling and other public policy domains has been fuelled by corporate support for individual-frame interventions focusing on individual behaviours and actions. Such perspectives provide easier policy solutions for governments as they place onus on individual action and defer the need for more systemic interventions that might be more politically unpalatable for some.^13^ The influence of behavioural sciences and their inadvertent^13^ and corporate-supported^23^ backing of individual-frame approaches has substantially facilitated this process. The 2023 *Lancet* Series on commercial determinants of health has provided ample evidence of these processes in various public health areas.^23^ Gambling, a field that has for a long time been characterised by strong commercial interests, is no exception.^24^, ^25^

To date, few systematic attempts have been made to map the extent to which individual-frame and system-frame perspectives are embedded within gambling legislation worldwide, and to examine how these perspectives are changing. Gambling policy research focuses strongly on country-level or jurisdiction-level descriptive studies, but comparative research and research on agenda setting and framing are largely missing. Our paper addresses this gap by conducting a global review of legislative change in gambling policy from 2018 to 2021. Our objectives were to map the global availability of legal forms of gambling, the changes in its provision, and the prevalence of gambling harm prevention policies (stage 1); and to identify whether health and wellbeing is a focus within changing gambling legislation and, using critical frame analysis (CFA), to explore the extent to which individual-frame and system-frame perspectives can be observed (stage 2).

To meet our objectives, we analysed written laws and regulations (recognising their crucial role as legal determinants of health)^26^ by following an approach used by public health policy surveillance projects.^27^ The policy cycle consists of several stages, ranging from agenda-setting to implementation and evaluation. The focus of the analysis presented here is on policy adoption, as articulated in written legislation. Analysis of policy cycle stages preceding and succeeding these policy outputs, and related questions about agenda-setting actors and policy implementation and effectiveness,^28^ are beyond the scope of this report.

## Methods

### Stage 1: global review

To systematically map global trends in gambling policy, we first conducted a review of legislative and regulatory changes between 2018 and 2021 for all countries, using the VIXIO Gambling Compliance database (ie, a dataset that monitors global gambling markets and regulations).^29^ We chose 2018 as the starting point as this year marked a shift in the framing of gambling as a public health issue at the global level. In 2018, WHO categorised gambling disorder within the 11th revision of the International Classification of Diseases under substance use and related disorders, recognising similarities with other addiction disorders to which public health perspectives and responses are typically applied.^30^ Our choice of inclusion dates also allowed us to focus on the most recent trends, especially among jurisdictions permitting gambling for the first time.

VM and JN carried out initial inductive coding of the VIXIO database for all available countries and territories. At this stage, we noted any legislative and regulatory changes introduced between 2018 and 2021, and the nature of these changes (eg, major legislative changes, such as extending or limiting legal provision of gambling; and other regulatory changes, such as limiting the location of gambling venues, restricting gambling advertising and marketing, raising the legal age of gambling, and introducing spending or loss limits, or the use of player data for prevention activity; appendix pp 12–21). The resulting coding was quality checked by additional web scraping by DU and HW. For the USA and Canada (which were not included in our VIXIO database licence), state-level web scraping was done and results were checked with regional experts.

### Stage 2: critical frame analysis

#### Case selection

**Figure 1:**
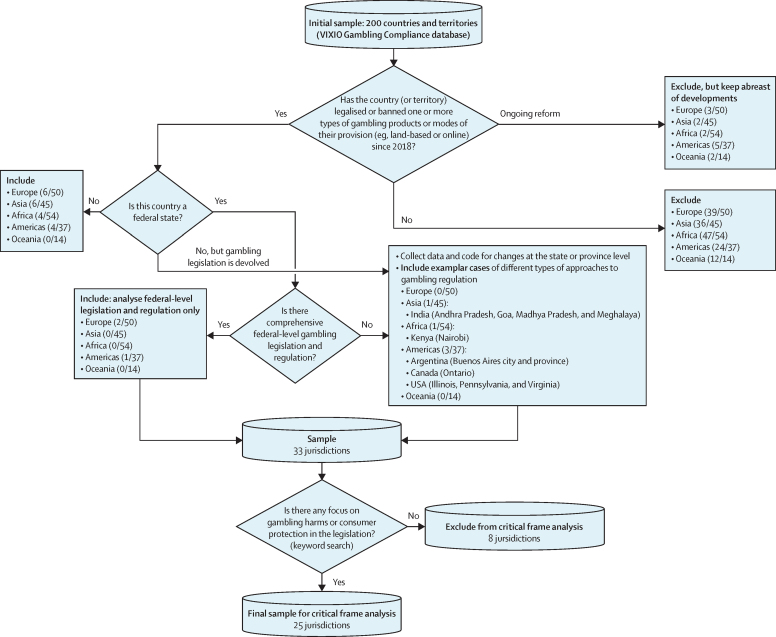
Sample selection decision tree

On the basis of the global mapping results, we identified all jurisdictions that had implemented major gambling legislation change between 2018 and 2021. A major change was defined as either legalisation of, or ban of, one or more types of gambling or modes of their provision (eg, land-based or online), or both. To our knowledge, this is the first analysis of this kind. As such, we restricted our scope by focusing on jurisdictions experiencing major legislative change rather than on those changing their regulation and regulatory controls, which is often done without primary legislative change. The case selection process is outlined in figure 1. In federal countries with no comprehensive federal-level gambling policy, state-level inclusion followed context-specific principles. In Argentina, Canada, India, and Kenya, we included individual states in which a major change had taken place. In the USA, most states had witnessed major changes in provision to expand the forms of gambling allowed. To avoid over-representation in the CFA, we included the states with the largest population within three different types of provision change: those now allowing online sports betting and online casinos (Pennsylvania); those allowing online sports betting only (Illinois); and those allowing new land-based and online gambling (Virginia).

#### Data extraction

Once cases were selected, policy documents were identified and extracted for systematic review and coding (appendix p 2). We searched government databases and regulator websites and conducted additional web searches to identify relevant documents. To ensure comparability across cases, we focused on primary gambling-related legislation and secondary legislation (including regulations specifically focused on addressing gambling-related harms) that were passed in 2018–21. Temporary regulations introduced during the COVID-19 pandemic were excluded from the analysis. Collectively, coauthors searched and coded documents in English, French, German, Italian, Portuguese, Russian, Spanish, Swedish, and Ukrainian. In jurisdictions where an English translation was not available (eg, Albania, Cambodia, Japan, Kosovo, the Netherlands, and Viet Nam), searches, coding, and analysis were done jointly with colleagues recommended by co-chairs of the *Lancet Public Health* Commission on Gambling.^4^

#### Keyword search and further case selection for critical frame analysis

Our objective for stage 2 was to explore the extent to which health and wellbeing is being considered within current gambling legislation. Keyword searches of all extracted documents were used to establish whether they included any focus on gambling harms or consumer protection. Search terms (translated for each language) were: “health”, “problem*”, “disordered”, “pathological”, “harm*”, “addict*”, “responsible”, “young*” OR “youth” OR “child*” OR “minor*”, “advertis*”, “marketing”, “consumer*”, and “protect*” (excluding data protection clauses). Legislative documents with these terms were included in the CFA (figure 1).

#### Critical frame analysis

We used CFA to code and analyse the selected documents.^31^, ^32^ CFA is a policy analysis method suitable for qualitative, large-N studies, which has been applied to public health policy analysis.^33^ CFA uncovers how particular meanings of reality (eg, gambling harms) are constructed in policy documents and how they shape proposed actions. CFA focuses on establishing the following dimensions of policy frames: (1) the diagnosis of a problem (ie, what is wrong?), (2) the attribution of causality (ie, who or what is responsible for the problem?), (3) the prognosis (ie, what should be done?), and (4) the call for action (ie, who should do something?).^32^ Given our exclusive focus on legislation and a high proximity between the prognosis and call for action categories in this type of policy document, we analyse and discuss them jointly (ie what should be done, and who should do this?).

Using a set of sensitising questions (panel), we coded each policy text to identify the policy frame (or frames) that underpin the legislation (appendix pp 25–26). An additional coding task was included under the prognosis category, in which we identified several different types of gambling harm prevention activities using a predefined codebook. The sensitising questions and the codebook were developed through a conceptual literature review.^34^, ^35^ Coding was piloted on four cases initially. All authors reviewed results and made amendments to the codebook based on the inductive findings. This pilot process ensured that harm prevention policy measures not captured in the initial framework were accounted for in the final version. Cases were allocated to each coauthor on the basis of their language knowledge and regional expertise. DU managed communication with the external consultants. Each policy document was coded twice by either two of the coauthors or jointly by DU and the external consultants. Disagreements about code use were resolved through a discussion among the coauthors. To facilitate cross-case analysis and comparisons, findings for each jurisdiction were summarised with a standardised template (appendix pp 10–11). All coding and analysis were done with Atlas.ti Web22.^36^

Panel **Critical frame analysis questions**

**Diagnosis (what is wrong?)**

• How is the nature of gambling addiction or gambling-related harms identified?
• Is the desire to gamble framed as natural?
• Are harms framed as the problem of a small (and stable) proportion of players?
• Is gambling framed as safe for the majority of players?
• Is the continuum of gambling-related harms recognised?
• Are harms understood as only individual harms? Or are social and population-level harms also considered?

**Attribution of causality (who and what is responsible for the problem?)**

• What, if any, is identified as a key cause or risk factor of gambling addiction or gambling-related harms?
• Individual neurobiological or psychological predispositions?
• Belonging to vulnerable groups?
• Illegal or unregulated markets?
• Product availability?
• Product design?
• Marketing promotions and advertising?
• Social networks?
• Other causes?

**Prognosis and call for action (what should be done, and who should do this?)**

• Is the responsible gambling principle explicitly invoked?
• Who is considered responsible for harm prevention, and in what way?
• What policy measures are proposed to tackle gambling harms?
• Are these measures more supply-side or demand-side focused?
• Are these measures focused on addressing individual or structural causes of gambling harms?
• Do they target the whole population? Or only vulnerable groups (eg, underage bettors and at-risk players)?

**Figure 2:**
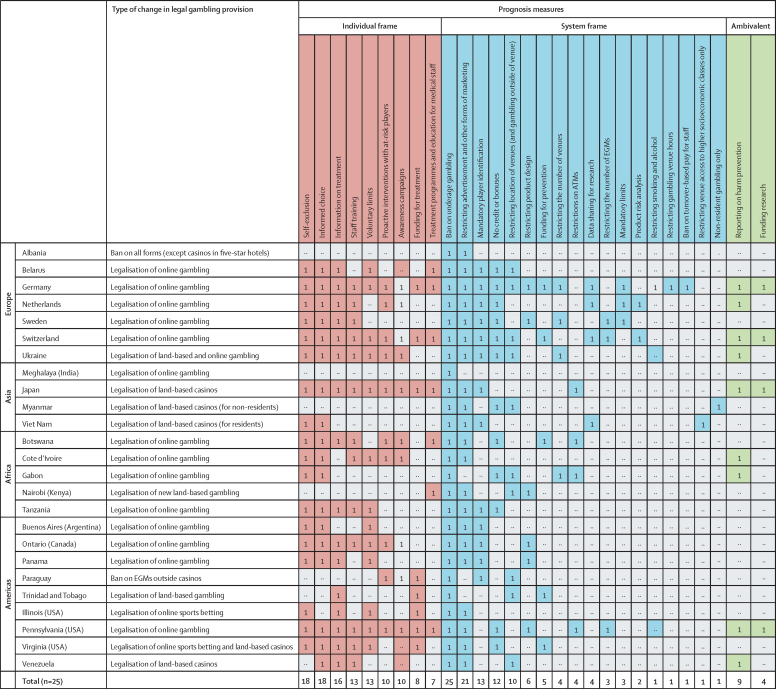
Individual-frame, system-frame, and ambivalent gambling harm prevention measures introduced in 25 jurisdictions with changes in legal gambling provision between 2018 and 2021 EGM=electronic gambling machine. Red=individual frame. Blue=system frame. Green=ambivalent measures that were classified as either individual frame or system frame depending on the jurisdiction context.

The final analytical step was to allocate identified prognosis codes to a policy frame by use of Chater and Loewenstein's individual-frame and system-frame taxonomy. All codes were listed and independently allocated to a policy frame by DU, VM, and HW, with broad agreement on coding. We classified prognosis codes into individual-frame, system-frame, and ambivalent (figure 2 and appendix pp 22–24). Ambivalent measures were those that could be defined as either individual frame or system frame depending on the context of their administration.

## Results

### Global gambling policy review

Legal gambling is widely available worldwide. More than 80% of countries (164 of 193) permit some form of gambling (including lotteries; table and appendix pp 12–21). Gambling is legally available in almost every European country (except Vatican City) and pan-American country (except Cuba). In Asia, legal gambling is available in 28 of 45 (61%) countries. 67 of 193 countries (35%) worldwide introduced some form of legislative or regulatory change for gambling between 2018 and 2021, the majority of which are European. The degree of change varies by region; European jurisdictions have introduced comparatively more restrictive regulations. For example, since 2018, 21 (43·8%) of 48 European jurisdictions have introduced some limitations on gambling advertising and marketing, compared with 3 (11·1%) of 27 in Asia; however, Asia also contains the highest number of countries that do not legally permit gambling.

**Table:** Global trends in gambling legislation and regulation between 2018 and 2021

	Countries where gambling is legislated at the country or federal level*	**Any legal gambling**	**Any kind of legislative or regulatory change**	**Introduced (or reintroduced) regulatory controls for legal gambling**				
				Restricting locations of gambling venues	Restricting advertisement and other forms of marketing	Changes to player data handling requirements	Legal age raised	New or stricter spending or loss limits
Europe (50 countries)	49 (100·0%)	48 (98·0%)	36 (73·5%)	12 (24·5%)	21 (42·9%)	14 (28·6%)	4 (8·2%)	4 (8·2%)
Asia (45 countries)	44 (100·0%)	27 (61·4%)	12 (27·3%)	1 (2·3%)	3 (6·8%)	2 (4.5%)	..	..
Africa (54 countries)	53 (100·0%)	46 (86·8%)	10 (18·9%)	3 (5·7%)	6 (11·3%)	1 (1·9%)	..	..
Americas (37 countries)	34 (100·0%)	33 (97·1%)	8 (23·5%)	3 (8·8%)	3 (8·8%)	1 (2·9%)	..	..
Oceania (14 countries)	13 (100·0%)	10 (76·9%)	1 (7·7%)	..	..	..	..	..
World (200 countries)	193 (100·0%)	164 (85·0%)	67 (34·7%)	19 (9·8%)	33 (17·1%)	18 (9·3%)	4 (2·1%)	4 (2·1%)

* Seven countries were excluded from the overview based on this criterion: Bosnia and Herzegovina (Europe); India (Asia); Kenya (Africa); Argentina, Canada, and the USA (Americas); and Australia (Oceania). In these countries, gambling is legislated primarily at the state, province, or regional level. Inclusion of jurisdiction-level data from these countries was considered unfeasible, as it would skew the results. Source: VIXIO Gambling Compliance database^29^ with additional web scraping conducted by the research team.

### Critical frame analysis

33 jurisdictions in which major legislative changes were introduced between 2018 and 2021 were identified (appendix pp 3–5), including eight from Europe, ten from Asia, five from Africa, and ten from the Americas. Of these 33 cases, 26 had extended legal provision and seven had introduced bans. The jurisdictions banning gambling included two from Europe, four from Asia, and one from the Americas. 25 (75·8%) of 33 jurisdictions with major legislative change included some focus on health and consumer protection measures within their policies and were thus included in the CFA.

#### Diagnosis of gambling-related harms: what is wrong?

Harmful gambling was recognised as a potentially serious health and wellbeing issue in most jurisdictions (21/25). However, individual framing was predominant and system framing of gambling-related harms was almost absent. In all cases, individual-level harms were foregrounded compared with harms to concerned significant others and wider social and economic harms. 17 jurisdictions (68%) referred to addiction directly in their legislative texts. Negative consequences or harms were also used in 16 (64%) of 25 jurisdictions, but these terms were generally mentioned in passing. Exceptions included Japan and the Netherlands, who provided a more precise definition of these harms, including financial issues, social isolation, suicide, poverty, and crime. Apart from addiction, numerous alternative individual-level terms were used for diagnosing the problem, such as excessive gambling; compulsive gambling (or gamblers); problem gambling, gamblers, or bettors; pathological gambling; high-risk players; or at-risk gamblers. Multiple jurisdictions had discursive juxtapositions of so-called problem gambling and responsible gambling (or, as in the US state of Illinois, problem gambling *vs* responsible gaming).^37^ Dutch regulations on remote gambling start with the following definitions: “problem players: players whose gaming behaviour presents a high risk of gambling addiction due to a persistent and increasing inability to resist the urge to play”, and “recreational players: players whose gaming behaviour shows little or no addiction potential”.^38^

There were some exceptions to this individual framing. In Germany, the legislation focused strongly on how increasing the availability of gambling products could increase the risks of addiction and harm in the population. Japan's Basic Action Plan on Gambling Addiction highlighted multiple harms at the individual, family, and societal levels, including debt, crime, poverty, child abuse, and suicide.^39^

#### Framing the causes of gambling-related harms: who or what is responsible?

The discussion of causes in the 25 legislative texts analysed was mostly limited (14), unelaborated, and arguably relied on conceptions of the so-called irresponsible, addicted, or problem individual. When causes of harms were explicitly discussed, they were mainly framed at the system level. In six (24%) of 25 cases, illegal gambling was identified as a potential cause of gambling harms. For example, the Dutch Remote Gambling Act framed the need for legalisation in the following way: “a responsible, reliable, and verifiable offer of games of chance is made possible by setting strict requirements for a license to offer remote games of chance. In this way the licensed offer can be separated from illegal offer of which it is not clear whether it is responsible and reliable”.^40^ In four (16%) of 25 texts, the availability of gambling was identified as a potential cause of harmful gambling. This cause was discussed in jurisdictions introducing a ban on some forms of gambling (eg, Paraguay), but also in cases where the provision of legal gambling expanded (eg, Germany, Myanmar, and Pennsylvania in the USA). Potentially harmful effects of gambling marketing, that were thought to incite excessive participation (according to the Netherlands' policy rules) were recognised in some jurisdictions (7 [28%] of 25 texts). Another supply-side factor considered to be the cause of gambling harms in some jurisdictions was gambling product design (10 [40%] of 25 texts). Finally, legislation in Ontario (Canada) included an explicit recognition that operator practices could cause harm: “players [may be] allowed to play excessively by operators”.^41^

#### Prognosis and call for action: framing ways to address gambling-related harms

Analysis of the prognosis codes showed that the focus for solutions was predominantly on individuals and individual control, although a few cases did recognise the operators' responsibilities towards players. Responsible gambling was used as a term by 18 (72%) of the 25 analysed texts. By contrast, “duty of care” was only used by two jurisdictions (Sweden and the Netherlands).

So-called responsible gambling and the measures being proposed under its umbrella are in line with individual framing. For example, Tanzanian Internet Gaming regulations stated that “the responsible gaming policy shall contain the following: (a) information on problem gaming and a link to that information; (b) information of customer service center with internal support team to handle player with problem gaming; (c) a list of player protection measures that are available on the site and access to these measures; (d) a link to a simple self-assessment process to determine a risk potential; (e) information and links to the Board's website”.^42^ These common provisions were included in many of the texts analysed.

In terms of system framing, Sweden and the Netherlands explicitly included an operator's duty of care in their legislation, and eight other jurisdictions mandated it in some other form (40·0%). However, how the operators' responsibility to players was conceptualised varied between jurisdictions. In many cases, strong individual framing was still retained, as shown in the Swedish Gambling Act: “A licensee shall ensure that social and health considerations are observed in the gambling activities in order to protect players against excessive gambling and help them to reduce their gambling where there is a reason to do so (duty of care)”.^43^ The Netherlands' Remote Gambling Act highlights similar individual framing: “2.2.1. The license holder who organises remote games of chance (as do operators of land-based casinos and gaming arcades) has an active duty of care to help the player as much as possible in taking their own responsibility”.^40^

Coding prognosis measures according to their framing (ie, individual frame, system frame, or ambivalent) showed the dominance of individual-frame measures (figure 2). The most frequently encountered individual-frame measures were self-exclusion (18 of 25; 72%), so-called informed choice measures (18 of 25; 72%), information on treatment (16 of 25; 64%), staff training (13 of 25; 52%), voluntary limits (13 of 25; 52%), and interventions with at-risk players (10 of 25; 40%). Measures also included technologically enabled solutions, such as mandatory statements to players (5 of 25; 20%) to facilitate informed choice, and electronic systems for player behaviour monitoring (5 of 25; 20%) to identify and intervene with those thought to be at risk of harm. Although such systems put onus on the operator to review player behaviour, the focus is still on managing how individuals play rather than changing the structural environment of gambling provision. Generic information campaigns to raise public awareness of gambling harms were common in the sample (8 of 25). In Belarus and Japan, these campaigns included school-based programmes. Notable attention was also given to the treatment of gambling addiction across the analysed countries. Measures included funding treatment (8 of 25; 32%) and treatment programmes or training for medical staff (7 of 25; 28%). Further explanation of the categorisation of these measures can be found in the appendix (pp 22–24).

System-frame measures were less prevalent and less elaborated in the policy texts than individual-frame measures. The only universally applied system-frame measure was a ban on underage gambling. Another widely adopted system-frame measure was restricting advertisement and other forms of gambling marketing (21 of 25; 84%). These restrictions varied in severity and ranged from stringent requirements, such as a ban on all sports betting advertisement in Albania (a ban that was later lifted in January, 2023) to simple statements that advertising without authorisation from the regulator was not allowed (Argentina). Restrictions on marketing primarily focused on so-called vulnerable groups, such as underage people (15 of 21; 71%) and self-excluded or at-risk players (12 of 21; 57%). Mandatory player identification was required in half of the sampled jurisdictions (13 of 25; 52%). Few jurisdictions introduced restrictions reducing population exposure to gambling (6 of 21; 29%). Other system-level measures included: restrictions on provision of bonuses or credit (12 of 25; 57%), restrictions on the location of venues (10 of 25; 40%), restrictions on product design (6 of 25; 24%), funding for prevention (5 of 25; 20%), mandating data sharing by operators for research purposes (4 of 25; 16%), restrictions on access to ATMs (4 of 25; 16%); restrictions on the total number of venues allowed in a jurisdiction, as well as the number of venues in a given area (4 of 25; 16%), restrictions on the number of electronic gambling machines per venue (3 of 25; 12%), mandatory limits (3 of 25; 12%), mandatory risk analysis of games (2 of 25; 8%), restricting smoking and alcohol in venues (2 of 25; 8%), restricting the operational hours of gambling venues (1 of 25; 4%), restricting venue entry to high socioeconomic classes only (1 of 25; 4%), allowing non-resident gambling only (1 of 25; 4%), and a ban on turnover-based pay for staff (1 of 25; 4%).

We identified two ambivalent prognosis measures that aligned more closely with individual framing in some jurisdictions and with system framing in others. Although operators in many countries are obliged to report on the effectiveness of gambling harm prevention actions (9 of 25; 36%), important variations exist in what and how they are expected to report. Conducting and funding research on gambling addiction (4 of 25; 16%) was placed into the ambivalent category.

A heatmap (figure 2) shows that individual-frame measures, such as self-exclusion, informed choice, signposting to treatment, voluntary limit-setting, staff training, and interventions with at-risk players typically cluster together. Individual-frame clusters are observed across multiple jurisdictions and are particularly typical of the jurisdictions that have legalised online gambling. Strong clusters of system-frame measures, on the contrary, are present in few European jurisdictions only (Germany, Sweden, and Switzerland). These system-frame clusters are largely formed by measures focusing on restricting the availability of land-based gambling, such as restricting the number or location of venues or electronic gambling machines.

## Discussion

Gambling is legally available in most countries worldwide and is becoming increasingly legislated. Our examination of countries introducing major legislative changes between 2018 and 2021 also highlighted regional differences. In Europe, legislation introduced greater regulatory restrictions, and a somewhat greater (although not universal) focus on system-frame approaches. However, in countries such as the USA, where the focus is on legalising markets for the first time, a greater dominance of individual framing of the problem and its solutions is apparent. The difference is likely to be related to market maturity, especially in online gambling. We found that changing the provision of online gambling is often accompanied with a greater focus on individual—rather than systemic—prevention measures.

Almost all the reviewed legislative texts simultaneously included elements of both individual and system frames, especially within their proposed measures. On the surface, this approach suggests an acknowledgment that reducing and preventing gambling harm requires multimodal and multilevel interventions.^44^ However, the conceptualisation of harms was narrow and the diagnosis of harms had an overwhelming focus on the individual's gambling addiction. Consideration of broader ecosystems of corporate, political, and economic actors and circumstances that generate harms were typically not considered. Harms were commonly framed as only affecting the minority of individuals deemed to be irresponsible (eg, so-called problem gamblers) and not the majority of recreational players viewed as responsible. Little recognition was given to the continuum of gambling harms or harms to others, despite evidence of these harms in public health-oriented gambling research.^45^, ^46^ Although a minority of jurisdictions included a focus on some of the more visible structural causes of gambling-related harms (eg, product availability, marketing, and venue locations), most of the proposed gambling harm prevention measures were strongly influenced by individual-frame approaches. The concept of responsible gambling was embedded within many policies, highlighting individual responsibility and treatment of people afflicted by so-called problem gambling as a primary response to perceived challenges.

Exceptions to the predominance of individual-frame approaches consist of measures restricting the availability of land-based gambling, marketing-related restrictions, and an emerging focus on operators' duty of care to bettors. Restrictions on the availability and marketing of gambling could reflect a reaction to public health concerns raised in research and by the broader public, who are increasingly exposed to such marketing.^9^, ^47^ Although the implementation of policies was beyond the scope of this paper, a qualitative reading suggests that their scope varies to an important degree. Some jurisdictions took a population-based approach to regulating gambling advertisement exposure, but more commonly restrictions tended to focus on protecting so-called vulnerable groups, such as underage people and self-excluded and at-risk players. Furthermore, duty of care was conceptualised as encouraging players to be responsible (as seen in the Netherlands).

A few countries, such as Sweden and Germany, have a comparatively comprehensive adoption of system-frame harm prevention measures. This adoption is an interesting development which, in our data, is only visible in the European region. These jurisdictions represent potentially promising examples of system-level prevention. However, the effectiveness of their policies in terms of public health goals depends on implementation, which requires careful evaluation and monitoring. In Germany, a longitudinal 3-year evaluation of gambling-related harm prevention under the 2021 Gambling Treaty will be led by independent public health researchers and began on July 10, 2023.^48^

A system-level public health approach to gambling is not yet translating into comprehensive policy action across jurisdictions. Gambling research and policies have only recently considered public health approaches and have tended to rely on a rather insular intervention paradigm,^18^, ^24^ which is arguably replicated in the legislative texts we reviewed. Given that the efficacy of many individual-frame measures in gambling has been queried,^9^, ^49^, ^50^, ^51^ and that they have been shown to yield small or even null results in other public policy areas,^13^ many jurisdictions currently reforming their gambling policies rely on weak solutions to gambling-related harms. Although many individual-frame measures, such as treatment, are necessary under any configuration, they should not replace preventive system-frame actions. The predominance of individual-frame measures in legislation restricts future possibilities for public health action on gambling-related harms. When gambling is made legal, the responsibility for preventing harms lies not only with providers and gamblers, but also with legislators and regulators who permit gambling within their jurisdiction and govern all characteristics associated with its provision.

Our study has limitations. Data for the first round of case selection were drawn from the VIXIO Gambling Compliance database, which provides information of interest to industry actors. We therefore inherit any bias from this data source (eg, an increased focus on new markets and news reporting on European, North American, and Australian jurisdictions). We conducted additional web scraping to address this potential bias. Regarding the CFA, some of the policy documents were very brief—particularly when gambling bans were introduced—and we were not able to access some preparatory documents. We also did not include policy documents that were in draft form, such as sports betting regulations in Brazil. This exclusion could limit the documentation of public health-oriented framings within the sample. Conversely, our case selection of US states probably underestimates the predominance of individual-frame approaches. Our exclusion of countries without comprehensive federal-level gambling legislation from the initial policy review could underestimate our assessment of the breadth of legal gambling provision globally. Future research could reapply our analytical approach to jurisdictions such as the USA or India on a state-by-state basis. Finally, our sample selection criteria imply that our findings reflect trends in newly liberalising markets and could be less representative of more mature ones. Further research on a bigger sample of jurisdictions, including those that witnessed substantial regulatory shifts (without changes in the legal provision of gambling), could address these limitations.

Our focus has been to analyse legislative documents. This analysis has not extended to reviewing how these legislative texts were formulated. Given the powerful role of commercial actors in public health policy framing,^23^ future research should also focus on the agenda-setting and policy formulation stages of the policy cycle. Addressing the implementation or efficacy of these policies was also beyond the scope of our project. Whether planned measures are actually implemented and the extent of their implementation are important topics for future research. Further qualitative research into the content of policies, such as advertisement or duty of care, would also be needed to assess whether they are targeted at protecting the public health or the vulnerable few. However, understanding the legal determinants of health is an essential first step, as this framing governs subsequent actions.^26^

Global policy surveillance is a powerful public health tool.^27^, ^52^ This study highlights a disparity between increased recognition of the public health harms associated with gambling and their translation into policy change, especially in newly legalising countries. This disparity has implications for how gambling harms are treated and prevented and can ultimately affect the protection of public health.

## Search strategy and selection criteria

To inform our sensitising questions and coding framework, we conducted a conceptual literature review focusing on how the debate about the RENO model and responsible gambling versus public health approaches to preventing gambling harms has evolved since 1999. Our goal was to explore the underlying assumptions about gambling-related harms, their causes, and proposed policies and interventions in this scholarship, with a particular focus on differences and tensions between the approaches. We searched PubMed, PsycINFO, and Business Source Ultimate with keywords in the title or abstract: “gambling” AND (“public health” OR “responsible gambling” OR “RENO”) for papers published between Jan 1, 1999, and Dec 31, 2021, in English. Searches yielded 547 (PubMed), 640 (PsycINFO), and 210 (Business Source Ultimate) results. We reviewed abstracts of all publications and selected the most relevant conceptual and review-style papers for full-text review and analysis. We also conducted forward and backward citations searches of the selected publications. A total of 78 full-text publications were reviewed (appendix pp 6–9).

## Declaration of interests

DU has been funded as a member of staff at the University of Glasgow to work on this project by the Wellcome Trust through a Humanities and Social Sciences Fellowship to HW. VM has received funding from the Finnish Ministry of Social Affairs and Health (section 52 of the Finnish Lotteries Act), the Academy of Finland (project 349589 CODEG; Project 31834 POLEG), the Finnish Foundation for Alcohol Studies, and French Observatory for Drugs and Drug Addiction. VM has been paid for delivering a webinar by Bochum University and for peer reviews by Routledge. VM has received support for travel from the Finnish Foundation of Alcohol Studies. VM is a member of the Gambling Harms Evaluation committee of the Finnish Ministry of Social Affairs and Health and has provided expert advice and consultations to third sector and public sector actors in Finland. JN has been funded by the Finnish Ministry of Social Affairs and Health. This funding emanates from the gambling monopoly in Mainland Finland, based on section 52 in the Finnish Lotteries Act (1047/2001), which stipulates that gambling issues must be researched. JN has also received funding from the Academy of Finland (project 349589; Commercial Determinants of Harm in the Digital Environment). JN has received support for travel from the Finnish Foundation of Alcohol Studies. JN has obtained consultancy fees and travel support from governmental actors, private sector companies (including The Recycling Lottery, regulated under the Lotteries Act in Finland), and non-governmental organisations to provide insights on gambling-related harm. HW has received funding for gambling-related projects from the National Institute for Health Research, Economic and Social Research Council, Wellcome Trust, Office of Health Improvements and Disparities, Public Health England, Gambling Commission (including from regulatory settlements), Gambling Research Exchange Ontario, Greater London Authority, Greater Manchester Combined Authority, and the Department for Culture Media and Sport. HW has received funding from GambleAware for a project on gambling and suicide; consultancy fees from the Institute of Public Health, Ireland and the National Institute for Economic and Social Research; payment for her role as Deputy Chair of the Advisory Board for Safer Gambling, remunerated by the Gambling Commission; payment as an expert witness on gambling by Lambeth and Middlesborough Borough Councils; payment for delivery of a webinar by McGill University; has provided unpaid advice on research to GamCare; has received support for travel from the Turkish Green Crescent Society, Gambling Regulators European Forum, and Alberta Gambling Research Institute; is a member of the WHO Panel on gambling; and runs a research consultancy practice for public and third sector bodies. HW has not, and does not, provide services to the gambling industry.
