# Supplementary material for: Public health approaches to gambling: a global review of legislative trends
Source: Lancet Public Health. 2023 Nov 7;9(1):e57–67. doi: 10.1016/S2468-2667(23)00221-9 (PMC10927617; doi:10.1016/S2468-2667(23)00221-9)
Supplement: Supplementary appendix [file mmc1.pdf]

THE LANCET
Public Health

Supplementary appendix

This appendix formed part of the original submission and has been peer reviewed. We post it as supplied by the authors.

Supplement to: Ukhova D, Marionneau V, Nikkinen J, Wardle H. Public health approaches to gambling: a global review of legislative trends. *Lancet Public Health* 2023; published online Nov 6. https://doi.org/10.1016/S2468-2667(23)00221-9.

Embracing public health approaches to gambling? A review of global legislative and regulatory trends

Authors: Daria Ukhova PhD,[1] Virve Marionneau PhD,[2] Janne Nikkinen PhD,[2] Prof. Heather Wardle PhD[1]

[1] School of Social and Political Sciences, University of Glasgow, Glasgow, United Kingdom, [2] Centre for Research on Addiction, Control and Governance (CEACG), University of Helsinki, Helsinki, Finland

SUPPLEMENT

TABLE OF CONTENTS

FIGURE S1: DOCUMENT SELECTION DECISION TREE **2**

TABLE S1: OVERVIEW OF CASES INCLUDED IN STAGE 2 **3**

TABLE S2: CONCEPTUAL LITERATURE REVIEW: BIBLIOGRAPHY **6**

TABLE S3: STANDARDISED CASE SUMMARY TEMPLATE **10**

TABLE S4: GLOBAL MAPPING: COUNTRY-LEVEL DATA, 2018-2021 **12**

TABLE S5: CRITICAL FRAME ANALYSIS: FINAL CODING FRAMEWORK AND CODE FREQUENCIES (N=25) **22**

TABLE S6: A SNAPSHOT OF CRITICAL FRAME ANALYSIS AS APPLIED IN THIS PAPER **25**

Figure S1: Document selection decision tree

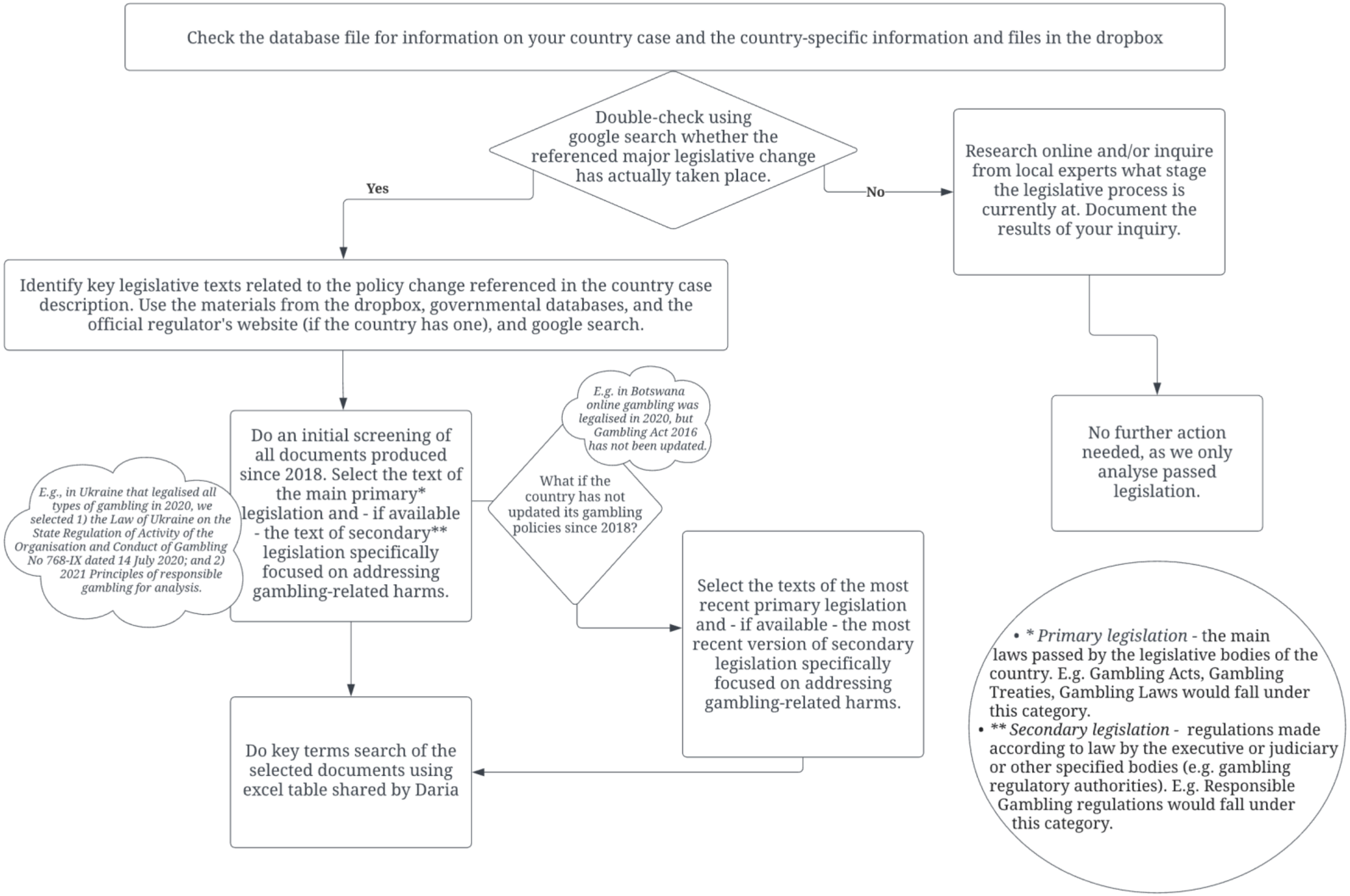

Table S1: Overview of cases included in Stage 2

Countries	Major legislative change 2018-2021	Phase 1 (*document selection + keywords search*): Included data sources	Phase 2 (*CFA*)
**Europe**		**8 cases**	**7 cases**
Albania	2018 ban on all forms of gambling (except casinos in 5-star hotels)	• Law no. 75/2018 on Some additions and amendments to Law no. 155/2015, "On games of chance in the Republic of Albania"	Included
Belarus	2018 legalisation of online gambling	• Order of the President of the Republic of Belarus 'On the improvement of legal regulation of the gambling business' no. 305 from 7 August 2018<br>• Resolution of the Council of Ministers of the Republic of Belarus from 1 March 2019 no.139 'On measures of realisation of the Order of President of the Republic of Belarus' no.305 from 7 August 2018<br>• Action plan on minimisation of negative impacts of games of chance on the population of the Republic of Belarus 2021-2022<br>• Law of the Republic of Belarus on changes to advertisement laws no.82-3 from 4 January 2021	Included
Germany (federal system)	2020 legalisation of online gambling	• State Treaty on the re-regulation of gaming in Germany (State Treaty on Gaming 2021 – GlüStV 2021)	Included
Kosovo	2019 ban on all forms of gambling	• 2019 Law no.06/L-155 on the prohibition of gambling	Excluded
Netherlands	2021 legalisation of online gambling	• Decree from 26 January 2021 to conform the implementation of the Remote Gambling Act (KOA)<br>• Regulation of the Minister for Legal Protection from 21 January 2021, no. 3181155, containing provisions for the implementation of the Remote Gambling Act (Remote Gambling Regulations)<br>• 2021 Policy rules of the Board of Directors of the Gambling Authority on responsible gambling	Included
Sweden	2019 legalisation of online gambling	• Gambling Act 2018: 1138	Included
Switzerland (federal system)	2019 legalisation of online gambling at the federal level	• Federal Law on Gambling from 29 September 2017<br>• Ordinance on Gambling from 7 November 2018<br>• Ordinance of the Swiss Confederation Casino Commission on casinos from 7 November 2018	Included
Ukraine	2020 re-legalisation of land-based and online gambling (banned in 2009)	• The Law of Ukraine on the State Regulation of Activity of the Organisation and Conduct of Gambling No 768-IX from 14 July 2020<br>• Principles of responsible gambling 11 August 2021	Included
**Asia**		**10 cases**	**4 cases**
Bangladesh	2020 ban on all forms of gambling	• Written Petition No. 15090 of 2016 (Judgment dated 10.02.2020)	Excluded
Cambodia	2019 ban on online gambling	• 2019 Sub-decree creating department for personal safety and fight against commercial gambling<br>• 2020 Law on the Management of Integrated Resorts and Commercial Gambling (LMCG)	Excluded
India (federal system)			
○ Andhra Pradesh	2020 ban on online gambling	• Andhra Pradesh Gaming (Amendment) Act, 2020	Excluded
○ Goa	2020 ban on casino gambling for residents	• Goa Public Gambling (Amendment) Act, 2021	Excluded
○ Madhya Pradesh	2021 re-legalisation of lottery (banned in 1993)	• Gazette notification to resume the sale of lottery tickets as well as commence gambling and betting starting August 23 2021	Excluded

**Countries**	**Major legislative change 2018-2021**	**Phase 1 (*document selection + keywords search*): Included data sources**	**Phase 2 (*CFA*)**
○ Meghalaya	2021 legalisation of online gambling	• The Meghalaya Regulation of Gaming Act, 2021 (Act No. 9 of 2021)	Included
Japan	2018 legalisation of land-based casinos	• Act on Development of Specified Integrated Resort Districts (Act no. 80 from 27 July 2018)<br>• Basic law on measures against gambling addiction (Act no.74 from 13 July 2018)<br>• 2022 Basic plan for promoting measures against gambling addiction	Included
Myanmar	2019 legalisation of land-based casinos (for foreigners only)	• Gambling Law 13/2019	Included
Uzbekistan	2020 legalisation of sports betting	• Order of the President of the Republic of Uzbekistan on 'Measures to raise football to a completely new stage of development in Uzbekistan' from 4 December 2019	Excluded
Vietnam	2019-2024 pilot legalisation of land-based casinos for Vietnam residents	• No 03/2017/ND-CP Decree on casino business	Included
**Africa**		**5 cases**	**5 cases**
Botswana	2020 legalisation of online gambling	• Botswana gambling regulations 2016<br>• Botswana gambling act 2012	Included
Cote D'Ivoire	2020 legalisation of online gambling	• Legal regime for games of chance (Law no. 2020-480 from 27 May 2020)	Included
Gabon	2020 legalisation of online gambling	• Ordinance No. No. 0012/PR/2020 of 08/14/2020 regulating games for entertainment, money and chance in the Gabonese Republic<br>• Law No. 040/2020 of 02/18/2021 ratifying Ordinance No. 0012/PR/2020 of August 14, 2020 regulating games for entertainment, money and chance in the Gabonese Republic	Included
Kenya (Nairobi only) (devolved gambling legislation)	2021 legalisation of new types of land-based gambling (betting, gaming, totalizator, and new country-wide lottery)	• Nairobi City County Betting, Lotteries and Gaming Act, 2021	Included
Tanzania	2021 online gambling legalisation	• Gambling Act 2003 (still valid and complemented by the internet gaming regulations)<br>• Internet gaming regulations 2021	Included
**Americas**		**10 cases included**	**9 cases**
Argentina (federal system):<br>○ Buenos Aires (Autonomous City of Buenos Aires & Province of Buenos Aires)	2019 legalisation of online gambling	• Law 15,079/18 from 11 December 2018<br>• Decree 181/19 from 25 March 2019	Included
Brazil (federal system)	2018 legalisation of fixed odds sports betting	• Law 13.756 from 12 December 2018	Excluded
Canada (federal system)<br>○ Ontario	2021 legalisation of single-event sports betting at the federal level<br>2022 legalisation of online sports betting and casino ('new igaming market') in Ontario	• Bill C-218 (Act to amend the Criminal Code (sports betting) from 29 June 2021<br>• 1992 Ontario Gaming Control Act (still valid, last amended in 2020)<br>• 2022 Registrar's Standards for Internet Gaming	Included

**Countries**	**Major legislative change 2018-2021**	**Phase 1 (*document selection + keywords search*): Included data sources**	**Phase 2 (*CFA*)**
Panama	2020 legalisation of online gambling for residents	• Resolution no.11 from 6 March 2020	Included
Paraguay	2021 ban on non-casino EGMs	• Presidential note CONAJZAR 725/2020<br>• Law 6903 that establishes measures to protect minor age from influences of EGMs from 20 April 2022	Included
Trinidad & Tobago	2021 legalisation of land-based gambling	• Gambling (Gaming and Betting) Control Act no.8 2021	Included
United States (federal system)			
○ Illinois	2019 legalisation of online sports betting	• 230 ILCS 10/ Gambling Act<br>• 2019 230 ILCS 45/ Sports Wagering Act.<br>• Illinois Administrative Code. TITLE 11: Alcohol, horse racing, lottery, and video gaming subtitle: Sports wagering. Chapter 1: Illinois Gaming Board. Part 1900 Sports Wagering: Sections 1900.340 Advertising and marketing; 1900.1230 Client requirements; 1900.1250 Responsible gaming limits; 1900.1730 Duties of licenses regarding self-excluded persons; 1900.1660 Signage; 1900.1220 Sport wagering accounts	Included
○ Pennsylvania	2018 legalisation of online gambling (sports betting and casino)	• 2017 Act 42 amending Agriculture code (3 PA.C.S.) and Amusements (4 PA.C.S.) of the Pennsylvania Consolidates Statutes	Included
○ Virginia	2020 legalisation of online sports betting and land-based casinos	• Virginia 2020 Sports Betting Bill 384 (as approved on 22 April 2020)<br>• Virginia Administrative Code. Chapter 80. Sports Betting Consumer Protection Program.	Included
Venezuela	2021 re-legalisation of land-based casinos	• 1997 Law for the control of casinos, bingo halls and slot machines<br>• 1998 Regulation of the Law for the control of casinos, bingo halls and slot machines<br>• 2007 Law on taxes on betting or gambling activities<br>• 2010 Administrative ruling no.058 establishing the obligation to report, in casinos and bingo halls, on gambling-related harms<br>• 2019 Administrative Ruling no. DE-19-01, which establishes the rules for the prevention, control and inspection of money laundering and terrorism financing crimes, which must be implemented by casinos and bingo halls	Included

Table S2: Conceptual literature review: Bibliography

To inform the development of sensitizing questions and coding framework, we reviewed 78 papers published between 1999 and 2021. These were conceptual publications on RENO model, 'responsible gambling', and public health discourses/frames/approaches to gambling-related harms; and review-style papers on the effectiveness of various policies and interventions. The latter were included to ensure the comprehensiveness of the 'prognosis' part (proposed measures) of our coding framework. Publications are presented in chronological order. Alphabetical order is used within individual years, except for the cases when there were several related publications in the same year (e.g., responses to an article). The latter are sequenced chronologically.

Year of publication	Title
1999	Korn DA, Shaffer HJ. Gambling and the health of the public: adopting a public health perspective. *J Gambl Stud* 1999; **15**: 289–365.
2002	Shaffer HJ, Korn DA. Gambling and related mental disorders: A public health analysis. *Annu Rev Public Health* 2002; **23**: 171–212.
2003	Korn D, Gibbins R, Azmier J. Framing public policy towards a public health paradigm for gambling. *J Gambl Stud* 2003; **19**: 235–56.<br>Shaffer HJ. A public health perspective on Gambling: The four principles. *AGA Responsible Gaming Lect Ser* 2003; **2**: 1–28.
2004	Blaszczynski A, Ladouceur R, Shaffer HJ. A Science-Based Framework for Responsible Gambling: The Reno Model. *J Gambl Stud* 2004; **20**: 301–17.<br>Messerlian C, Derevensky J, Gupta R. A public health perspective for youth gambling. *Int Gambl Stud* 2004; **4**: 147–60.
2005	Messerlian C, Derevensky J, Gupta R. Youth gambling problems: A public health perspective. *Health Promot Int* 2005; **20**: 69–79.
2007	Livingstone C, Woolley R. Risky Business: A Few Provocations on the Regulation of Electronic Gaming Machines. *Int Gambl Stud* 2007; **7**: 361–76.<br>Reith G. Gambling and the contradictions of consumption: A genealogy of the 'pathological' subject. *Am Behav Sci* 2007; **51**: 33–55.
2008	Blaszczynski A, Ladouceur R, Nower L, Shaffer H. Informed Choice and Gambling: Principles for Consumer Protection. *J Gambl Bus Econ* 2008; **2**: 103–18.<br>Dickson-Gillespie L, Rugle L, Rosenthal R, Fong T. Preventing the incidence and harm of gambling problems. *J Prim Prev* 2008; **29**: 37–55.<br>Reith G. Reflections on Responsibility. *J Gambl Issues* 2008; **22**: 149.
2009	Adams PJ, Raeburn J, De Silva K. A question of balance: Prioritizing public health responses to harm from gambling. *Addiction* 2009a; **104**: 688–91.<br>Adams PJ, Raeburn J, De Silva K. Gambling beneficiaries having their cake and eating it: The attractions of avoiding responsible gambling regulation. *Addiction* 2009b; **104**: 697–8.<br>Griffiths MD. Minimizing harm from gambling: What is the gambling industry's role? *Addiction* 2009; **104**: 696–7.<br>Marshall D. Gambling as a public health issue: The critical role of the local environment. *J Gambl Issues* 2009; : 66.<br>Mcdonald J. The biggest challenge? Recognition of gambling as a public issue. *Gambl Res* 2009; **21**: 47–50.
2010	Bogart WA. Permit But Discourage. Oxford: Oxford University Press, 2010 DOI:10.1093/acprof:oso/9780195379877.001.0001.<br>Livingstone C, Woolley R, Keleher H. Productivity commission inquiry into Australia's gambling industry. Canberra: Productivity Commission, 2010.
2011	Blaszczynski A, Collins P, Fong D, *et al.* Responsible Gambling: General Principles and Minimal Requirements. *J Gambl Stud* 2011; **27**: 565–73.

Year of publication	Title
2012	Planzer S, Wardle H. What we know about the comparative effectiveness of gambling regulation. *Eur J Risk Regul* 2012; **3**: 410–5.<br><br>Williams RJ, West BL, Simpson RI. Prevention of Problem Gambling: A Comprehensive Review of the Evidence, and Identified Best Practices. Report prepared for the Ontario Problem Gambling Research Centre and the Ontario Ministry of Health and Long Term Care. October 2012.
2013	Gainsbury SM, Blankers M, Wilkinson C, Schelleman-Offermans K, Cousijn J. Recommendations for international gambling harm-minimisation guidelines: Comparison with effective public health policy. *J Gambl Stud* 2013; **30**: 771–88.<br><br>Orford J. Power, Powerlessness and Addiction. Cambridge, UK: Cambridge University Press, 2013 DOI:10.1017/CBO9781139540971.
2014	Gainsbury SM. Review of Self-exclusion from Gambling Venues as an Intervention for Problem Gambling. *J Gambl Stud* 2014; **30**: 229–51.<br><br>Livingstone C, Rintoul A, Francis L. What is the evidence for harm minimisation measures in gambling venues? *Evid Base* 2014; **2014**: 1–24.<br><br>Planzer S, Gray HM, Shaffer HJ. Associations between national gambling policies and disordered gambling prevalence rates within Europe. *Int J Law Psychiatry* 2014; **37**: 217–29.
2015	Collins P, Blaszczynski A, Ladouceur R, *et al.* Responsible Gambling: Conceptual Considerations. *Gaming Law Rev Econ* 2015; **19**: 594–9.
2016	Browne M, Langham E, Rawat V, *et al.* Assessing gambling-related harm in Victoria. Melbourne: Victorian Responsible Gambling Foundations. 2016.<br><br>Hancock L, Hao Z. Gambling regulatory regimes and the framing of "responsible gambling" by transnational casino corporations: Asia-Pacific regimes in comparative perspective. *Asia Pacific J Public Adm* 2016; **38**: 139–53.<br><br>Ladouceur R, Blaszczynski A, Shaffer HJ, Fong D. Extending the Reno Model: Responsible Gambling Evaluation Guidelines for Gambling Operators, Public Policymakers, and Regulators. *Gaming Law Rev Econ* 2016; **20**: 580–6.<br><br>Langham E, Thorne H, Browne M, Donaldson P, Rose J, Rockloff M. Understanding gambling related harm: A proposed definition, conceptual framework, and taxonomy of harms. *BMC Public Health* 2016; **16**. DOI:10.1186/s12889-016-2747-0.<br><br>Shaffer HJ, Ladouceur R, Blaszczynski A, Whyte K. Extending the RENO Model: Clinical and Ethical Applications. *Am J Orthopsychiatry* 2016; **86**: 297–309.
2017	Delfabbro P, King D. Prevention paradox logic and problem gambling: Does low-risk gambling impose a greater burden of harm than high-risk gambling? *J Behav Addict* 2017; **6**: 163–7.<br><br>Browne M, Rockloff MJ. The dangers of conflating gambling-related harm with disordered gambling. *J Behav Addict* 2017; **6**: 317–20.<br><br>Hancock L, Smith G. Critiquing the Reno Model I-IV International Influence on Regulators and Governments (2004–2015)— the Distorted Reality of "Responsible Gambling". *Int J Ment Health Addict* 2017; **15**: 1151–76.<br><br>Abbott MW. Beyond Reno: a Critical Commentary on Hancock and Smith. *Int J Ment Health Addict* 2017; **15**: 1177–86.<br><br>Delfabbro P, King DL. Blame It on Reno: a Commentary on Hancock and Smith. *Int J Ment Health Addict* 2017; **15**: 1203–8.<br><br>Shaffer HJ, Blaszczynski A, Ladouceur R. Truth, Alternative Facts, Narrative, and Science: What Is Happening to Responsible Gambling and Gambling Disorder? *Int J Ment Health Addict* 2017; **15**: 1197–202.<br><br>Young M, Markham F. Rehabilitating Reno: a Commentary on Hancock and Smith. *Int J Ment Health Addict* 2017; **15**: 1187–92.<br><br>Hancock L, Smith G. Replacing the Reno Model with a Robust Public Health Approach to "Responsible Gambling": Hancock and Smith's Response to Commentaries on Our Original Reno Model Critique. *Int J Ment Health Addict* 2017; **15**: 1209–20.<br><br>Ladouceur R, Shaffer P, Blaszczynski A, Shaffer HJ. Responsible gambling: a synthesis of the empirical evidence. *Addict Res Theory* 2017; **25**: 225–35.<br><br>Hellman M, Örnberg JC, Livingstone C. Gambling policy studies: a field that is growing in size and complexity. *Addict Res Theory* 2017; **25**: 433–5.<br><br>Tanner J, Drawson AS, Mushquash CJ, Mushquash AR, Mazmanian D. Harm reduction in gambling: a systematic review of industry strategies. *Addict Res Theory* 2017; **25**: 485–94.

Year of publication	Title
	Rintoul A, Deblaquiere J, Thomas A. Responsible gambling codes of conduct: lack of harm minimisation intervention in the context of venue self-regulation. *Addict Res Theory* 2017; **25**: 451–61.<br>The Lancet. Problem gambling is a public health concern. *Lancet* 2017; **390**: 913.<br>Veselka L, Pradeep B, Hudson R, Turner NE. A public health approach to gambling: A report prepared for Gambling Research Exchange Ontario (GREO). London, Ontario, 2017.
2018	Abbott M, Binde P, Clark L, *et al.* Conceptual Framework of Harmful Gambling, Third Edition. Guelph, Ontario, Canada, 2018 DOI:10.33684/CFHG3.en.<br>Browne M, Rockloff MJ. Prevalence of gambling-related harm provides evidence for the prevention paradox. *J Behav Addict* 2018; **7**: 410–22.<br>Chóliz M. Ethical Gambling: A Necessary New Point of View of Gambling in Public Health Policies. *Front Public Heal* 2018; **6**: 1–5.<br>Hing N, Russell AMT, Hronis A. A definition and set of principles for responsible consumption of gambling. *Int Gambl Stud* 2018; **18**: 359–82.<br>Wardle H, Reith G, Best D, Mcdaid D, Platt S. Measuring gambling-related harms: a framework for action. Birmingham, UK: Gambling Commission. 2018.
2019	Delfabbro P, King DL. Challenges in the Conceptualisation and Measurement of Gambling-Related Harm. *J Gambl Stud* 2019; **35**: 743–55.<br>Livingstone C, Rintoul A, de Lacy?Vawdon C, et al. Identifying effective policy interventions to prevent gambling?related harm. 2019. https://responsiblegambling.vic.gov.au/resources/ publications/identifying?effective?policy?interventions?to?prevent? gambling?related?harm?640/ (accessed April 20, 2023)<br>McMahon N, Thomson K, Kaner E, Bambra C. Effects of prevention and harm reduction interventions on gambling behaviours and gambling related harm: an umbrella review. Addict Behav 2019; 90: 380–88.<br>Reith G, Wardle H, Gilmore I. Gambling harm: a global problem requiring global solutions. *Lancet* 2019; **394**: 1212–4.<br>Shaffer HJ, Blaszczynski A, Ladouceur R, Fong D, Collins P, editors. Responsible Gambling: Primary Stakeholder Perspectives. Oxford: Oxford University Press, 2019 DOI:10.1093/med-psych/9780190074562.001.0001.<br>Sulkunen P, Babor TF, Cisneros Ornberg J, *et al.* Setting Limits. Oxford: Oxford University Press, 2019 DOI:10.1093/oso/9780198817321.001.0001.<br>van Schalkwyk MCI, Cassidy R, McKee M, Petticrew M. Gambling control: in support of a public health response to gambling. *Lancet* 2019; **393**: 1680–1.<br>Wardle H, Reith G, Langham E, Rogers RD. Gambling and public health: We need policy action to prevent harm. *BMJ* 2019; **365**: 1–5.
2020	Christensen DR. Effective harm minimisation practices: Public Health Implications. In: Bowden-Jones H, Dickson C, Dunand C, Simon O, eds. Harm reduction for gambling: A public health approach. London and New York: Routledge, 2020: 78–90.<br>Christensen DR. Responsible Gambling: Who is Responsible? *Crit Gambl Stud* 2020. DOI:10.29173/cgs83.<br>Delfabbro P, King DL. On the Limits and Challenges of Public Health Approaches in Addressing Gambling-Related Problems. *Int J Ment Health Addict* 2020; **18**: 844–59.<br>Hilbrecht M, Baxter D, Abbott M, *et al.* The conceptual framework of harmful gambling: A revised framework for understanding gambling harm. *J Behav Addict* 2020; **9**: 190–205.<br>Livingstone C. Harm prevention and reduction efforts in gambling disorder. In: Bowden-Jones H, Dickson C, Dunand C, Simon O, eds. Harm reduction for gambling: A public health approach. London and New York: Routledge, 2020: 91–101.<br>Livingstone C, Rintoul A. Moving on from responsible gambling: a new discourse is needed to prevent and minimise harm from gambling. *Public Health* 2020; **184**: 107–12.<br>Reynolds J, Kairouz S, Ilacqua S, French M. Responsible Gambling: A Scoping Review. *Crit Gambl Stud* 2020; **1**: 23–39.<br>Shaffer HJ, Blaszczynski A, Ladouceur R. Gambling Control and Public Health: Let's Be Honest. *Int J Ment Health Addict* 2020; **18**: 819–24.<br>Abbott M. Gambling Control and Public Health: Let's Be Really Honest. *Int J Ment Health Addict* 2020; **18**: 825–34.

Year of publication	Title
2021	Akçayır M, Nicoll F, Baxter DG, Palmer ZS. Whose Responsibility Is It to Prevent or Reduce Gambling Harm? A Mapping Review of Current Empirical Research. *Int J Ment Health Addict* 2021. DOI:10.1007/s11469-020-00459-x.<br><br>Blank L, Baxter S, Woods HB, Goyder E. Interventions to reduce the public health burden of gambling-related harms: a mapping review. *Lancet Public Health* 2021; **6**: e50–63.<br><br>Blaszczynski A, Shaffer HJ, Ladouceur R, Collins P. Clarifying Responsible Gambling and its Concept of Responsibility. *Int J Ment Health Addict* 2021. DOI:10.1007/s11469-020-00451-5.<br><br>Browne M, Rawat V, Tulloch C, Murray-Boyle C, Rockloff M. The evolution of gambling-related harm measurement: Lessons from the last decade. *Int J Environ Res Public Health* 2021; **18**. DOI:10.3390/ijerph18094395.<br><br>Delfabbro P, Parke J, Dragecvic S, Percy C, Bayliss R. Safer by design: Building a collaborative, integrated and evidence-based framework to inform the regulation and mitigation of gambling product risk. *J Gambl Issues* 2021; **48**: 158–201.<br><br>Marionneau V, Egerer M, Nikkinen J. How Do State Gambling Monopolies Affect Levels of Gambling Harm? *Curr Addict Reports* 2021; **8**: 225–34.<br><br>Price A, Hilbrecht M, Billi R. Charting a path towards a public health approach for gambling harm prevention. *J Public Health (Bangkok)* 2021; **29**: 37–53.<br><br>Shaffer HJ, Ladouceur R. Moving Away from Individual Responsibility: A Comment. *J Gambl Stud* 2021; **37**: 1071–8.<br><br>van Schalkwyk MCI, Petticrew M, Cassidy R, *et al.* A public health approach to gambling regulation: countering powerful influences. *Lancet Public Health* 2021; **6**: e614–9.

Table S3: Standardised case summary template

Country (region/state)					
Selection criteria (why the country has been included?)					
Regulatory regime (introduced by the legislation)					
Product types covered by the legislation	Lottery	Casinos	Sports betting	EGMs	Online
Regulator					
Legislative and regulatory documents included in the analysis					
'Diagnosis' (what is wrong)<br>How is the nature of gambling addiction and/or gambling-related harms identified?<br>Is desire to gamble framed as 'natural'?<br>Are harms framed as a problem of a small (and stable) proportion of players?<br>Is gambling framed as safe for the majority of players?<br>Is there a recognition of the continuum of gambling-related harms?<br>Are harms understood as only individual harms? Or are social and population level harms also considered?					
Attribution of causality (Who/what is responsible for the problem?)<br>What/if any is identified as key causes of gambling addiction and/or gambling-related harms?<br>• Individual psychological/neurobiological predisposition?<br>• Belonging to 'vulnerable' groups?<br>• Illegal/unregulated market?<br>• Product design?<br>• Product availability?<br>• Marketing promotions, advertising?<br>• Social networks?<br>• Other?					

Prognosis (What should be done?)<br>Is ‘responsible gambling’ principle explicitly invoked?<br>Who is considered responsible for harm prevention and in what way?<br>What policy measures are proposed to tackle gambling harms?<br>Are these measures more supply- or demand-side focused?<br>Are they focused on addressing individual or structural ‘causes’ of gambling harms?<br>Do they target the whole population? Or rather ‘vulnerable’ groups (e.g., minors, ‘at-risk’ players, etc.)?<br><br>Based on the results of coding policy measures, how would you evaluate which policy frame, individual-frame (RG) or system-frame (PH) appears more dominant? What type of measures (individual vs. population-based) are more elaborated/discussed in more detail?	
Any harmful actions proposed?	
Any examples of well-elaborated public health policies?	

Table S4: Global mapping: Country-level data, 2018-2021

**Region (number of countries)/ Country**	**Gambling legislated primarily at country-/federal level***	**Any legal gambling at all**	**Any kind of legislative or regulatory change**	**Major legislative change (i.e., legalising or banning one or more types of gambling products or modes of their provision)**	**Type of major legislative change**		**(Re-)introduced regulatory controls (legal gambling)**					**(Re-) introduced measures against illegal gambling**
					**Extending legal provision**	**Limiting legal provision**	**Limiting locations of gambling venues**	**Restricting advertisement and other forms of marketing**	**Changes to player data handling requirements**	**Legal age raised**	**New or stricter spending/ loss limits**	
**Europe (50)**												
Albania	1	1	1	1	··	1	1	1	··	··	··	1
Andorra	1	1	1	··	··	··	··	··	··	··	··	··
Armenia	1	1	1	··	··	··	1	1	··	··	··	1
Austria	1	1	··	··	··	··	··	··	··	··	··	··
Azerbaijan	1	1	··	··	··	··	··	··	··	··	··	··
Belarus	1	1	1	1	1	··	1	1	1	1	··	1
Belgium	1	1	1	··	··	··	··	1	··	··	1	··
Bosnia&Herzegovina (excluded)*	··	N/A	N/A	N/A	N/A	N/A	N/A	N/A	N/A	N/A	N/A	N/A
Bulgaria	1	1	1	··	··	··	1	··	1	··	··	··
Croatia	1	1	1	··	··	··	1	··	··	··	··	1
Cyprus	1	1	1	··	··	··	··	1	··	··	··	1
Czech Republic	1	1	1	··	··	··	··	··	1	··	··	··
Denmark	1	1	1	··	··	··	··	··	1	··	··	··
Estonia	1	1	··	··	··	··	··	··	··	··	··	··
Finland	1	1	1	··	··	··	··	··	1	··	1	1
France	1	1	1	··	··	··	··	··	1	··	··	··
Georgia	1	1	1	··	··	··	··	1	··	1	··	1
Germany	1	1	1	1	1	··	1	1	1	··	1	1
Greece	1	1	1	··	··	··	··	··	··	··	··	··
Hungary	1	1	1	··	··	··	··	··	··	··	··	··
Iceland	1	1	··	··	··	··	··	··	··	··	··	··
Ireland	1	1	1	··	··	··	··	1	··	1	··	··

Region (number of countries)/ Country	Gambling legislated primarily at country- /federal level*	Any legal gambling at all	Any kind of legislative or regulatory change	Major legislative change (i.e., legalising or banning one or more types of gambling products or modes of their provision)	Type of major legislative change		(Re-)introduced regulatory controls (legal gambling)					(Re-) introduced measures against illegal gambling
					Extending legal provision	Limiting legal provision	Limiting locations of gambling venues	Restricting advertisement and other forms of marketing	Changes to player data handling requirements	Legal age raised	New or stricter spending/ loss limits	
Italy	1	1	1	..	..	..	..	1	..	..	..	1
Kosovo	1	1	1	1	..	1	..	..	..	..	..	..
Latvia	1	1	1	..	..	..	1	1	1	1	..	1
Liechtenstein	1	1	1	..	..	..	..	..	..	..	..	..
Lithuania	1	1	1	..	..	..	..	1	..	..	..	..
Luxembourg	1	1	..	..	..	..	..	..	..	..	..	..
Malta	1	1	1	..	..	..	..	..	..	..	..	..
Moldova	1	1	1	..	..	..	..	1	..	..	..	..
Monaco	1	1	..	..	..	..	..	..	..	..	..	..
Montenegro	1	1	..	..	..	..	..	..	..	..	..	..
Netherlands	1	1	1	1	1	..	..	1	1	..	..	1
North Macedonia	1	1	..	..	..	..	..	..	..	..	..	..
Norway	1	1	..	..	..	..	..	..	..	..	..	..
Poland	1	1	1	..	..	..	1	..	..	..	..	1
Portugal	1	1	1	..	..	..	..	1	..	..	..	..
Romania	1	1	..	..	..	..	..	..	..	..	..	..
Russia	1	1	1	..	..	..	..	1	..	..	..	1
San Marino	1	1	..	..	..	..	..	..	..	..	..	..
Serbia	1	1	1	..	..	..	..	1	1	..	..	1
Slovakia	1	1	1	..	..	..	1	1	1	..	..	..
Slovenia	1	1	..	..	..	..	..	..	..	..	..	..
Spain	1	1	1	..	..	..	..	1	..	..	..	..
Sweden	1	1	1	1	1	..	1	1	1	..	..	1
Switzerland	1	1	1	1	1	..	1	1	1	..	..	1

Region (number of countries)/ Country	Gambling legislated primarily at country-/federal level*	Any legal gambling at all	Any kind of legislative or regulatory change	Major legislative change (i.e., legalising or banning one or more types of gambling products or modes of their provision)	Type of major legislative change		(Re-)introduced regulatory controls (legal gambling)					(Re-) introduced measures against illegal gambling
					Extending legal provision	Limiting legal provision	Limiting locations of gambling venues	Restricting advertisement and other forms of marketing	Changes to player data handling requirements	Legal age raised	New or stricter spending/ loss limits	
Turkey	1	1	1	··	··	··	··	··	··	··	··	1
Ukraine	1	1	1	1	1	··	1	1	1	··	··	1
United Kingdom (UK)	1	1	1	··	··	··	··	··	··	··	1	··
Vatican City (Holy See)	1	··	··	··	··	··	··	··	··	··	··	··
**Europe - Total**	**49**	**48**	**36**	**8**	**6**	**2**	**12**	**21**	**14**	**4**	**4**	**18**
as % of European countries included in mapping	*100·00*	*97·96*	*73·47*	*16·33*	*12·24*	*4·08*	*24·49*	*42·86*	*28·57*	*8·16*	*8·16*	*36·73*
**Asia (45)**												
Afghanistan	1	··	··	··	··	··	··	··	··	··	··	··
Bahrain	1	··	··	··	··	··	··	··	··	··	··	··
Bangladesh	1	1	1	1	··	1	··	··	··	··	··	1
Bhutan	1	1	··	··	··	··	··	··	··	··	··	··
Brunei	1	··	··	··	··	··	··	··	··	··	··	··
Cambodia	1	1	1	1	··	1	··	··	··	··	··	1
China	1	1	1	··	··	··	··	··	··	··	··	1
India (excluded)*	··	N/A	N/A	N/A	N/A	N/A	N/A	N/A	N/A	N/A	N/A	N/A
Indonesia	1	··		··	··	··	··	··	··	··	··	··
Iran	1	··	··	··	··	··	··	··	··	··	··	··
Iraq	1	··	··	··	··	··	··	··	··	··	··	··
Israel	1	1	··	··	··	··	··	··	··	··	··	··
Japan	1	1	1	1	1	··	··	1	1	··	··	1
Jordan	1	··	··	··	··	··	··	··	··	··	··	··
Kazakhstan	1	1	··	··	··	··	··	··	··	··	··	··

Region (number of countries)/ Country	Gambling legislated primarily at country-/federal level*	Any legal gambling at all	Any kind of legislative or regulatory change	Major legislative change (i.e., legalising or banning one or more types of gambling products or modes of their provision)	Type of major legislative change		(Re-)introduced regulatory controls (legal gambling)					(Re-) introduced measures against illegal gambling
					Extending legal provision	Limiting legal provision	Limiting locations of gambling venues	Restricting advertisement and other forms of marketing	Changes to player data handling requirements	Legal age raised	New or stricter spending/ loss limits	
Kuwait	1	··	··	··	··	··	··	··	··	··	··	··
Kyrgyzstan	1	1	··	··	··	··	··	··	··	··	··	··
Laos	1	1	··	··	··	··	··	··	··	··	··	··
Lebanon	1	1	··	··	··	··	··	··	··	··	··	··
Macau	1	1	··	··	··	··	··	··	··	··	··	··
Malaysia	1	1	··	··	··	··	··	··	··	··	··	··
Maldives	1	··	··	··	··	··	··	··	··	··	··	··
Mongolia	1	1	··	··	··	··	··	··	··	··	··	··
Myanmar	1	1	1	1	1	··	1	1	··	··	··	1
Nepal	1	1	1	··	··	··	··	··	··	··	··	1
North Korea	1	1	··	··	··	··	··	··	··	··	··	··
Oman	1	··	··	··	··	··	··	··	··	··	··	··
Pakistan	1	1	··	··	··	··	··	··	··	··	··	··
Palestine	1	··	··	··	··	··	··	··	··	··	··	··
Philippines	1	1	1	··	··	··	··	··	··	··	··	··
Qatar	1	··	··	··	··	··	··	··	··	··	··	··
Saudi Arabia	1	··	··	··	··	··	··	··	··	··	··	··
Singapore	1	1	1	··	··	··	··	··	··	··	··	··
South Korea	1	1	··	··	··	··	··	··	··	··	··	··
Sri Lanka	1	1	1	··	··	··	··	··	··	··	··	1
Syria	1	··	··	··	··	··	··	··	··	··	··	··
Taiwan	1	1	··	··	··	··	··	··	··	··	··	1
Tajikistan	1	1	1	··	··	··	··	··	··	··	··	··
Thailand	1	1	··	··	··	··	··	··	··	··	··	··

Region (number of countries)/ Country	Gambling legislated primarily at country-/federal level*	Any legal gambling at all	Any kind of legislative or regulatory change	Major legislative change (i.e., legalising or banning one or more types of gambling products or modes of their provision)	Type of major legislative change		(Re-)introduced regulatory controls (legal gambling)					(Re-) introduced measures against illegal gambling
					Extending legal provision	Limiting legal provision	Limiting locations of gambling venues	Restricting advertisement and other forms of marketing	Changes to player data handling requirements	Legal age raised	New or stricter spending/ loss limits	
Timor-Leste	1	··	··	··	··	··	··	··	··	··	··	··
Turkmenistan	1	1	··	··	··	··	··	··	··	··	··	··
United Arab Emirates (UAE)	1	··	··	··	··	··	··	··	··	··	··	··
Uzbekistan	1	1	1	1	1	··	··	··	··	··	··	··
Vietnam	1	1	1	1	1	··	··	1	1	··	··	··
Yemen	1	··	··	··	··	··	··	··	··	··	··	··
**Asia - Total**	**44**	**27**	**12**	**6**	**4**	**2**	**1**	**3**	**2**	**0**	**0**	**8**
as % of Asian countries included in mapping	*100·00*	*61·36*	*27·27*	*13·64*	*9·09*	*4·55*	*2·27*	*6·82*	*4·55*	*0·00*	*0·00*	*18·18*
**Africa (54)**												
Algeria	1	1	··	··	··	··	··	··	··	··	··	··
Angola	1	1	··	··	··	··	··	··	··	··	··	··
Benin	1	1	··	··	··	··	··	··	··	··	··	··
Botswana	1	1	1	1	1	··	··	1	··	··	··	··
Burkina Faso	1	1	··	··	··	··	··	··	··	··	··	··
Burundi	1	1	··	··	··	··	··	··	··	··	··	··
Cabo Verde	1	1	··	··	··	··	··	··	··	··	··	··
Cameroon	1	1	1	··	··	··	··	··	··	··	··	1
Central African Republic (CAR)	1	1	··	··	··	··	··	··	··	··	··	··
Chad	1	1	··	··	··	··	··	··	··	··	··	··
Comoros	1	1	··	··	··	··	··	··	··	··	··	··
Congo, Democratic Republic	1	1	··	··	··	··	··	··	··	··	··	··
Congo, Republic	1	1	··	··	··	··	··	··	··	··	··	··

Region (number of countries)/ Country	Gambling legislated primarily at country-/federal level*	Any legal gambling at all	Any kind of legislative or regulatory change	Major legislative change (i.e., legalising or banning one or more types of gambling products or modes of their provision)	Type of major legislative change		(Re-)introduced regulatory controls (legal gambling)					(Re-) introduced measures against illegal gambling
					Extending legal provision	Limiting legal provision	Limiting locations of gambling venues	Restricting advertisement and other forms of marketing	Changes to player data handling requirements	Legal age raised	New or stricter spending/ loss limits	
Cote d'Ivoire	1	1	1	1	1	··	··	1	··	··	··	1
Djibouti	1	1	··	··	··	··	··	··	··	··	··	··
Egypt	1	1	··	··	··	··	··	··	··	··	··	··
Equatorial Guinea	1	1	··	··	··	··	··	··	··	··	··	··
Eritrea	1	··	··	··	··	··	··	··	··	··	··	··
Eswatini	1	1	··	··	··	··	··	··	··	··	··	··
Ethiopia	1	1	··	··	··	··	··	··	··	··	··	··
Gabon	1	1	1	1	1	··	1	··	··	··	··	1
Gambia	1	1	··	··	··	··	··	··	··	··	··	··
Ghana	1	1	1	··	··	··	··	1	··	··	··	··
Guinea	1	1	··	··	··	··	··	··	··	··	··	··
Guinea-Bissau	1	1	1	··	··	··	1	··	··	··	··	··
Kenya (excluded)*	··	N/A	N/A	N/A	N/A	N/A	N/A	N/A	N/A	N/A	N/A	N/A
Lesotho	1	1	··	··	··	··	··	··	··	··	··	··
Liberia	1	1	··	··	··	··	··	··	··	··	··	··
Libya	1	··	··	··	··	··	··	··	··	··	··	··
Madagascar	1	1	··	··	··	··	··	··	··	··	··	··
Malawi	1	1	··	··	··	··	··	··	··	··	··	··
Mali	1	··	··	··	··	··	··	··	··	··	··	··
Mauritania	1	··	··	··	··	··	··	··	··	··	··	··
Mauritius	1	1	··	··	··	··	··	··	··	··	··	··
Morocco	1	1	··	··	··	··	··	··	··	··	··	··
Mozambique	1	1	··	··	··	··	··	··	··	··	··	··
Namibia	1	1	1	··	··	··	1	1	··	··	··	1

Region (number of countries)/ Country	Gambling legislated primarily at country-/federal level*	Any legal gambling at all	Any kind of legislative or regulatory change	Major legislative change (i.e., legalising or banning one or more types of gambling products or modes of their provision)	Type of major legislative change		(Re-)introduced regulatory controls (legal gambling)					(Re-) introduced measures against illegal gambling
					Extending legal provision	Limiting legal provision	Limiting locations of gambling venues	Restricting advertisement and other forms of marketing	Changes to player data handling requirements	Legal age raised	New or stricter spending/ loss limits	
Niger	1	1	··	··	··	··	··	··	··	··	··	··
Nigeria	1	1	··	··	··	··	··	··	··	··	··	··
Rwanda	1	1	··	··	··	··	··	··	··	··	··	··
Sao Tome and Principe	1	1	··	··	··	··	··	··	··	··	··	··
Senegal	1	1	1	··	··	··	··	1	··	··	··	··
Seychelles	1	1	··	··	··	··	··	··	··	··	··	··
Sierra Leone	1	1	··	··	··	··	··	··	··	··	··	··
Somalia	1	··	··	··	··	··	··	··	··	··	··	··
South Africa	1	1	··	··	··	··	··	··	··	··	··	··
South Sudan	1	··	··	··	··	··	··	··	··	··	··	··
Sudan	1	··	··	··	··	··	··	··	··	··	··	··
Tanzania	1	1	1	1	1	··	··	1	1	··	··	1
Togo	1	1	··	··	··	··	··	··	··	··	··	··
Tunisia	1	1	··	··	··	··	··	··	··	··	··	··
Uganda	1	1	··	··	··	··	··	··	··	··	··	··
Zambia	1	1	··	··	··	··	··	··	··	··	··	··
Zimbabwe	1	1	1	··	··	··	··	··	··	··	··	··
**Africa - Total**	**53**	**46**	**10**	**4**	**4**	**0**	**3**	**6**	**1**	**0**	**0**	**5**
as % of African countries included in mapping	*100·00*	*86·79*	*18·87*	*7·55*	*7·55*	*0·00*	*5·66*	*11·32*	*1·89*	*0·00*	*0·00*	*9·43*
**Americas (37)**												
Antigua and Barbuda	1	1	··	··	··	··	··	··	··	··	··	v
Argentina (exclude)*	··	N/A	N/A	N/A	N/A	N/A	N/A	N/A	N/A	N/A	N/A	N/A

Region (number of countries)/ Country	Gambling legislated primarily at country- /federal level*	Any legal gambling at all	Any kind of legislative or regulatory change	Major legislative change (i.e., legalising or banning one or more types of gambling products or modes of their provision)	Type of major legislative change		(Re-)introduced regulatory controls (legal gambling)					(Re-) introduced measures against illegal gambling
					Extending legal provision	Limiting legal provision	Limiting locations of gambling venues	Restricting advertisement and other forms of marketing	Changes to player data handling requirements	Legal age raised	New or stricter spending/ loss limits	
Bahamas	1	1	..	..	..	..	..	..	..	..	..	..
Barbados	1	1	..	..	..	..	..	..	..	..	..	..
Belize	1	1	..	..	..	..	..	..	..	..	..	..
Bermuda	1	1	1	..	..	..	..	1	..	..	..	..
Bolivia	1	1	..	..	..	..	..	..	..	..	..	..
Brazil	1	1	1	1	1	..	..	..	..	..	..	..
Canada (exclude)*	..	N/A	N/A	N/A	N/A	N/A	N/A	N/A	N/A	N/A	N/A	N/A
Chile	1	1	..	..	..	..	..	..	..	..	..	..
Colombia	1	1	1	..	..	..	..	..	..	..	..	1
Costa Rica	1	1	..	..	..	..	..	..	..	..	..	..
Cuba	1	0	..	..	..	..	..	..	..	..	..	..
Curacao	1	1	..	..	..	..	..	..	..	..	..	..
Dominica	1	1	..	..	..	..	..	..	..	..	..	..
Dominican Republic	1	1	..	..	..	..	..	..	..	..	..	..
Ecuador	1	1	..	..	..	..	..	..	..	..	..	..
El Salvador	1	1	..	..	..	..	..	..	..	..	..	..
Grenada	1	1	..	..	..	..	..	..	..	..	..	..
Guatemala	1	1	..	..	..	..	..	..	..	..	..	..
Guyana	1	1	..	..	..	..	..	..	..	..	..	..
Haiti	1	1	..	..	..	..	..	..	..	..	..	..
Honduras	1	1	..	..	..	..	..	..	..	..	..	..
Jamaica	1	1	..	..	..	..	..	..	..	..	..	..
Mexico	1	1	1	..	..	..	..	1	..	..	..	1
Nicaragua	1	1	..	..	..	..	..	..	..	..	..	..

Region (number of countries)/ Country	Gambling legislated primarily at country-/federal level*	Any legal gambling at all	Any kind of legislative or regulatory change	Major legislative change (i.e., legalising or banning one or more types of gambling products or modes of their provision)	Type of major legislative change		(Re-)introduced regulatory controls (legal gambling)					(Re-) introduced measures against illegal gambling
					**Extending legal provision**	**Limiting legal provision**	**Limiting locations of gambling venues**	**Restricting advertisement and other forms of marketing**	**Changes to player data handling requirements**	**Legal age raised**	**New or stricter spending/ loss limits**	
Panama	1	1	1	1	1	··	··	··	1	··	··	1
Paraguay	1	1	1	1	··	1	1	··	··	··	··	1
Peru	1	1	··	··	··	··	··	··	··	··	··	··
Saint Kitts and Nevis	1	1	··	··	··	··	··	··	··	··	··	··
Saint Lucia	1	1	··	··	··	··	··	··	··	··	··	··
Saint Vincent and the Grenadines	1	1	··	··	··	··	··	··	··	··	··	··
Suriname	1	1	··	··	··	··	··	··	··	··	··	··
Trinidad and Tobago	1	1	1	1	1	··	1	··	··	··	··	1
United States of America (USA) (exclude)	··	N/A	N/A	N/A	N/A	N/A	N/A	N/A	N/A	N/A	N/A	N/A
Uruguay	1	1	··	··	··	··	··	··	··	··	··	··
Venezuela	1	1	1	1	1	··	1	1	··	··	··	1
**Americas - Total**	**34**	**33**	**8**	**5**	**4**	**1**	**3**	**3**	**1**	**0**	**0**	**6**
as % of American countries included in mapping	*100·00*	*97·06*	*23·53*	*14·71*	*11·76*	*2·94*	*8·82*	*8·82*	*2·94*	*0·00*	*0·00*	*17·65*
**Oceania (14)**												
Australia (exclude)*	··	N/A	N/A	N/A	N/A	N/A	N/A	N/A	N/A	N/A	N/A	N/A
Fiji	1	1	··	··	··	··	··	··	··	··	··	··
Kiribati	1	1	··	··	··	··	··	··	··	··	··	··
Marshall Islands	1	··	··	··	··	··	··	··	··	··	··	··
Micronesia	1	1	··	··	··	··	··	··	··	··	··	··
Nauru	1	1	··	··	··	··	··	··	··	··	··	··
New Zealand	1	1	1	··	··	··	··	··	··	··	··	··

**Region (number of countries)/ Country**	**Gambling legislated primarily at country- /federal level***	**Any legal gambling at all**	**Any kind of legislative or regulatory change**	**Major legislative change (i.e., legalising or banning one or more types of gambling products or modes of their provision)**	**Type of major legislative change**		**(Re-)introduced regulatory controls (legal gambling)**					**(Re-) introduced measures against illegal gambling**
					**Extending legal provision**	**Limiting legal provision**	**Limiting locations of gambling venues**	**Restricting advertisement and other forms of marketing**	**Changes to player data handling requirements**	**Legal age raised**	**New or stricter spending/ loss limits**	
Palau	1	1	··	··	··	··	··	··	··	··	··	··
Papua New Guinea	1	1	··	··	··	··	··	··	··	··	··	··
Samoa	1	1	··	··	··	··	··	··	··	··	··	··
Solomon Islands	1	1	··	··	··	··	··	··	··	··	··	··
Tonga	1	··	··	··	··	··	··	··	··	··	··	··
Tuvalu	1	··	··	··	··	··	··	··	··	··	··	··
Vanuatu	1	1	··	··	··	··	··	··	··	··	··	··
**Oceania - Total**	**13**	**10**	**1**	**0**	**0**	**0**	**0**	**0**	**0**	**0**	**0**	0
as % of Oceanian countries included in mapping	*100·00*	*76·92*	*7·69*	*0·00*	*0·00*	*0·00*	*0·00*	*0·00*	*0·00*	*0·00*	*0·00*	*0·00*
**World (200) - Total**	**193**	**164**	**67**	**23**	**18**	**5**	**19**	**33**	**18**	**4**	**4**	**37**
as % of all countries included in mapping	*100·00*	*84·97*	*34·72*	*11·92*	*9·33*	*2·59*	*9·84*	*17·10*	*9·33*	*2·07*	*2·07*	*19·17*

Notes: *Seven countries excluded from the overview based on this criterion: Bosnia & Herzegovina (Europe); India (Asia); Kenya (Africa); Argentina, Canada, US (Americas); Australia (Oceania). N/A: Not applicable.
Source: Vixio Gambling Compliance Database.

Table S5: Critical frame analysis: Final coding framework and code frequencies (N=25)

Dimensions of policy frames	Coding categories	Albania	Belarus	Germany	Netherlands	Sweden	Switzerland	Ukraine	Meghalaya (India)	Japan	Myanmar	Vietnam	Botswana	Cote d' Ivoire	Gabon	Nairobi (Kenya)	Tanzania	Buenos Aires (Arg)	Ontario (Canada)	Panama	Paraguay	Trinidad & Tobago	Illinois (USA)	Pennsylvania (USA)	Virginia (USA)	Venezuela	**Total (N=25)**	%
		**Regions/Countries**																										
		**Europe**							**Asia**				**Africa**					**Americas**										
***Diagnosis* (What is wrong?)**	"Gambling addiction" referred to directly	1	1	1	1	··	1	1	··	1	··	··	1	1	1	1	1	··	··	··	1	1	··	1	1	1	**17**	*68·0*
	Definition of addiction if any	N/A	N/A	N/A	N/A	N/A	N/A	N/A	N/A	N/A	N/A	N/A	N/A	N/A	N/A	N/A	N/A	N/A	N/A	N/A	N/A	N/A	N/A	N/A	N/A	N/A	N/A	N/A
	Other terms used to refer to harmful gambling	N/A	N/A	N/A	N/A	N/A	N/A	N/A	N/A	N/A	N/A	N/A	N/A	N/A	N/A	N/A	N/A	N/A	N/A	N/A	N/A	N/A	N/A	N/A	N/A	N/A	N/A	N/A
	Are gambling-related harms referred to? In what way?	··	1	1	1	1	1	1	··	1	1	··	··	1	··	··	1	1	1	··	1	1	··	1	··	1	**16**	*64·0*
***Causes* (What, if any, is identified as key 'causes' or 'risk factors' of gambling addiction and/or gambling-related harms?)**	Product design	··	··	1	1	1	1	1	··	1	··	··	1	1	··	··	··	1	1	··	··	··	··	··	··	··	**10**	*40·0*
	Gambling marketing (including advertisement)	··	··	1	1	··	1	1	··	1	··	··	1	··	··	··	··	··	1	··	··	··	··	··	··	··	**7**	*28·0*
	Illegal gambling/'Black market'	··	··	1	1	··	··	··	··	1	··	··	··	··	··	··	··	1	··	··	··	··	··	1	1	··	**6**	*24·0*
	Availability of gambling	··	··	1	··	··	··	··	··	··	1	··	··	··	··	··	··	··	··	··	1	··	··	1	··	··	**4**	*16·0*
	Belonging to 'vulnerable' groups	··	··	··	1	··	··	··	··	··	··	··	··	1	··	··	··	··	··	··	··	1	··	··	··	··	**3**	*12·0*
	Comorbidity with other addictions	··	··	··	··	··	··	··	··	1	··	··	··	··	··	··	··	··	··	··	··	··	··	1	··	··	2	*8·0*
	Individual psychological predispositions	··	··	··	··	1	··	··	··	··	··	··	··	··	··	··	··	··	··	··	··	··	··	··	··	··	**1**	*4·0*
	Operators allowing players to play excessively (in vivo)	··	··	··	··	··	··	··	··	··	··	··	··	··	··	··	··	··	1	··	··	··	··	··	··	··	**1**	*4, ·0*
***Prognosis* (What should be done?) — Individual-frame**	(Self)-exclusion	··	1	1	1	1	1	1	··	1	··	1	1	1	1	··	1	1	1	1	··	··	1	1	1	··	**18**	*72·0*
	Informed choice	··	1	1	1	1	1	1	··	1	··	1	1	1	1	··	1	1	1	1	··	··	··	1	1	1	**18**	*72·0*
	<u>Subcode</u>: Mandatory statements to players	··	··	1	··	1	1	··	··	··	··	··	··	··	··	··	··	··	1	··	··	··	··	1	··	··	**5**	*20·0*
	Information on treatment	··	1	1	1	1	1	1	··	1	··	··	1	··	··	··	1	··	1	1	··	1	1	1	1	1	**16**	*64·0*
	Staff training	··	··	1	1	1	1	1	··	1	··	··	1	1	··	··	1	··	1	··	··	··	··	1	1	1	**13**	*52·0*
	Voluntary limits	··	1	1	··	··	1	1	··	1	··	··	··	1	··	··	1	1	1	1	··	··	1	1	1	··	**13**	*52·0*
	Pro-active interventions with 'at-risk' players	··	··	1	1	··	1	1	··	1	··	··	1	1	··	··	··	··	1	··	1	··	··	1	··	··	**10**	*40·0*

Dimensions of policy frames	Coding categories	Regions/Countries																										
		Europe							Asia				Africa					Americas										
		Albania	Belarus	Germany	Netherlands	Sweden	Switzerland	Ukraine	Meghalaya (India)	Japan	Myanmar	Vietnam	Botswana	Cote d' Ivoire	Gabon	Nairobi (Kenya)	Tanzania	Buenos Aires (Arg)	Ontario (Canada)	Panama	Paraguay	Trinidad & Tobago	Illinois (USA)	Pennsylvania (USA)	Virginia (USA)	Venezuela	**Total (N=25)**	%
	Subcode: Use of electronic systems for player behaviour monitoring	··	··	1	1	1	1	··	··	··	··	··	··	··	··	··	··	··	··	··	··	··	··	1	··	··	**5**	*20·0*
	Awareness campaigns	··	1	··	··	··	··	1	··	1	··	··	1	1	··	··	··	··	··	··	··	··	··	1	1	1	**8**	*32·0*
	Subcode: School-based programmes	··	1	··	··	··	··	··	··	1	··	··	··	··	··	··	··	··	··	··	··	··	··	··	··	··	**2**	*8·0*
	Funding for treatment	··	··	1	··	··	1	··	··	1	··	··	··	··	··	··	··	··	··	··	1	1	1	1	1	··	**8**	*32·0*
	Treatment programmes and education for medical staff	··	1	1	··	··	1	··	··	1	··	··	1	··	··	1	··	··	··	··	··	··	··	1	··	··	**7**	*28·0*
**System-frame**	Ban on minors/youth gambling	1	1	1	1	1	1	1	1	1	1	1	1	1	1	1	1	1	1	1	1	1	1	1	1	1	**25**	*100·0*
	Restricting marketing (incl. advertisement)	1	1	1	1	1	1	1	··	1	1	1	1	1	··	1	1	1	1	1	··	··	1	1	1	1	**21**	*84·0*
	Subcode: Targeted at protecting minors	1	1	1	1	1	1	1	··	1	··	··	1	1	··	1	··	··	1	··	··	··	1	1	1	··	**15**	*60·0*
	Subcode: Targeted at protecting self-excluded and 'at-risk' players	··	··	1	1	1	1	1	··	1	··	··	··	1	··	··	1	··	1	··	··	··	1	1	1	··	**12**	*48·0*
	Subcode: Targeted at whole population	1	··	1	··	··	··	1	··	··	1	1	··	··	··	··	··	··	··	··	··	··	··	··	··	1	**6**	*24·0*
	Mandatory player identification	··	1	1	1	1	1	1	··	1	··	1	··	··	··	··	1	1	1	1	1	··	··	··	··	··	**13**	*52·0*
	No credit / bonuses	··	1	1	1	1	1	1	··	··	1	··	1	··	1	··	1	··	··	··	··	··	··	1	1	··	**12**	*48·0*
	Restricting location of venues and gambling outside of gambling venues	··	1	1	··	··	1	1	··	··	1	··	··	··	1	1	··	··	··	··	1	1	··	··	··	1	**10**	*40·0*
	Limiting product design	··	··	1	··	1	··	··	··	··	··	··	··	··	··	1	··	··	1	1	··	··	··	1	··	··	**6**	*24·0*
	Funding for prevention	··	··	1	··	··	1	··	··	··	··	··	1	··	··	··	··	··	··	··	··	1	··	··	1	··	**5**	*20·0*
	Limiting number of venues	··	··	1	··	1	··	1	··	··	··	··	··	··	1	··	··	··	··	··	··	··	··	··	··	··	**4**	*16·0*
	Restrictions on ATMs	··	··	··	··	··	··	··	··	1	··	··	1	··	1	··	··	··	··	··	··	··	··	1	··	··	**4**	*16·0*
	Data sharing for research	··	··	1	1	··	1	··	··	··	··	1	··	··	··	··	··	··	··	··	··	··	··	··	··	··	**4**	*16·0*

Dimensions of policy frames		Coding categories	Regions/Countries																									
			Europe							Asia				Africa					Americas										
			Albania	Belarus	Germany	Netherlands	Sweden	Switzerland	Ukraine	Meghalaya (India)	Japan	Myanmar	Vietnam	Botswana	Cote d' Ivoire	Gabon	Nairobi (Kenya)	Tanzania	Buenos Aires (Arg)	Ontario (Canada)	Panama	Paraguay	Trinidad & Tobago	Illinois (USA)	Pennsylvania (USA)	Virginia (USA)	Venezuela	**Total (N=25)**	%
		Limiting number of EGMs per venue	··	··	··	··	1	1	··	··	··	··	··	··	··	··	··	··	··	··	··	··	··	··	1	··	··	**3**	*12·0*
		Mandatory limits	··	··	1	1	1	··	··	··	··	··	··	··	··	··	··	··	··	··	··	··	··	··	··	··	··	**3**	*12·0*
		Product risk analysis	··	··	··	1	··	1	··	··	··	··	··	··	··	··	··	··	··	··	··	··	··	··	··	··	··	**2**	*8·0*
		Limiting smoking /alcohol	··	··	··	··	··	··	1	··	··	··	··	··	··	··	··	··	··	··	··	··	··	··	1	··	··	**2**	*8·0*
		Limiting gambling venue hours	··	··	1	··	··	··	··	··	··	··	··	··	··	··	··	··	··	··	··	··	··	··	··	··	··	**1**	*4·0*
		Ban on turnover-based pay for staff	··	··	1	··	··	··	··	··	··	··	··	··	··	··	··	··	··	··	··	··	··	··	··	··	··	**1**	*4·0*
		Restricting venue access to higher socio-economic classes only	··	··	··	··	··	··	··	··	··	··	1	··	··	··	··	··	··	··	··	··	··	··	··	··	··	**1**	*4·0*
		Non-resident gambling only	··	··	··	··	··	··	··	··	··	1	··	··	··	··	··	··	··	··	··	··	··	··	··	··	··	**1**	*4,0*
	**Ambivalent**	Reporting on harm prevention	··	··	1	1	··	1	1	··	1	··	··	··	1	1	··	··	··	··	··	··	··	··	1	··	1	**9**	*36·0*
		Funding research	··	··	1	··	··	1	··	··	1	··	··	··	··	··	··	··	··	··	··	··	··	··	1	··	··	**4**	*16·0*
***Call for action* (Who should do it?)**		Responsible gambling' (in vivo)	··	1	1	1	1	··	1	··	··	··	1	1	1	1	1	1	1	1	1	··	1	1	1	1	··	18	*72·0*
		Mandating operators' responsibility to protect players	··	··	1	1	1	1	1	··	1	··	··	··	1	··	··	··	1	1	··	··	··	··	··	··	1	10	*40·0*
		Subcode: 'Duty of care' (in vivo)	··	··	··	1	1	··	··	··	··	··	··	··	··	··	··	··	··	··	··	··	··	··	··	··	··	2	*8·00*

Notes: N/A: not applicable. ATM: Automated teller machine. EGM: Electronic gambling machine.

Table S6: A snapshot of critical frame analysis as applied in this paper

Dimensions of policy frames	Policy frames					
	Individual-frame		Ambivalent		System-frame	
	**Key characteristics**	**Examples from policy texts**	**Key characteristics**	**Examples from policy texts**	**Key characteristics**	**Examples from policy texts**
***Diagnosis* (What is wrong?)**	Gambling ‘addiction’/’disorder’ affecting a minority of players, so-called ‘problem gamblers’	'Problem players: Players whose gaming behaviour presents a high risk of gambling addiction due to a persistent and increasing inability to resist the urge to play’ ‘Recreational Players: Players whose gaming behaviour shows little or no addiction potential.’ (Netherlands, Remote Gambling Regulations 2021).<br><br>"excessive gambling or pathological gambling - strong addiction or compulsive participation in gambling and betting despite the negative consequences or the desire to quit" (Cote D’Ivoire, Law on Legal Regime for Games of Chance 2020)	N/A	N/A	Gambling could be harmful<br>Harms could be wide-ranging spanning individual, family, and societal levels	‘[gambling] causes critical social problems, such as multiple loans, poverty, violence, suicide, crimes and so on’ (Japan, Basic Law on Measures Against Gambling Addiction 2018)<br><br>‘(…) the social and economic consequences of a gambling disorder, including debt, treatment costs, suicide, criminal behaviour, unemployment and domestic issues’ (Pennsylvania, 2017 Act 42 amending Amusements)
***Causes* (What, if any, is identified as key ‘causes’ or ’risk factors’ of gambling addiction and/or gambling-related harms?)**	Individual psychological predispositions and belonging to ‘vulnerable’ groups	‘groups of persons regarded as vulnerable in any case include minors, young adults, persons who exhibit characteristics of risky gaming behaviour, consumers with an intellectual disability and consumers who have or have had an addiction to games of chance’ (Netherlands, 2021 Policy rules)	N/A	N/A	Supply-side factors, including: illegal gambling; availability of gambling in general; the influence of marketing; and product design.	*Illegal gambling/Black market:* ‘A responsible, reliable and verifiable offer of games of chance is made possible by setting strict requirements for a license to offer remote games of chance. In this way the licensed offer can be separated from *illegal offer of which it is not clear whether it is responsible and reliable*.’ (Netherlands, Remote Gambling Act (KOA) 2021)<br><br>*Availability*: ‘The objectives of this law are as follows – […] (b) to prevent and restrict gambling *as it is hazardous to the socio-economic life of the citizens*’ (Myanmar, Gambling Law 2019)<br><br>*Advertisement:* ‘In *advertising* the casino business or casino facilities, any person must give consideration to its influence over persons under twenty years of age and *the relationship between the use of casino facilities and addiction to casino gaming* and strive not to advertise or solicit excessively’ (Japan, Act on Integrated Resort 2018)<br><br>*Product design: […]* a number of differentiated measures for the individual game forms have been defined *in light of their specific potential for addiction* […] (Germany, State Treaty on Gaming 2021)

Dimensions of policy frames	Policy frames					
	**Individual-frame**		**Ambivalent**		**System-frame**	
	**Key characteristics**	**Examples from policy texts**	**Key characteristics**	**Examples from policy texts**	**Key characteristics**	**Examples from policy texts**
Prognosis and Call for action **(What should be done? And who should do it?)**	Primary responsibility for harm prevention lies with an individual player. Operators have responsibility to support players to take it. Individual level prevention measures include:<br>- self-management tools (self-exclusion; voluntary limit setting);<br>- provision of information on personal patterns of consumption;<br>- signposting to treatment;<br>- operator implemented measures to identify 'at-risk' players;<br>- awareness campaigns and education programmes;<br>- allocation of funding for treatment of 'gambling addiction';<br>- development of specialized treatment services and educational programmes for medical staff.	'The decision provides for *a coherent package of measures that gives the player the opportunity to gain and maintain control over his gaming behaviour*. For example, before and during participation in games of chance, the *player is made aware of the associated risks*. When the player registers with a licensed remote gambling provider, he must complete a player profile in which he indicates his playing limits. During the game, the player is reminded of these limits when these limits are (or threaten to be) exceeded. The license holder who organizes remote games of chance (as do operators of gaming casinos and gaming arcades) has *an active duty of care to help the player as much as possible in taking his own responsibility*.' (Netherlands, Remote Gambling Act (KOA) 2021)<br><br>'The responsible gaming policy shall contain the following: (a) *information on problem gaming* and a link to that information; (b) information of customer service center with internal support team to handle player with problem gaming; (c) *a list of player protection measures* that are available on the site and access to these measures; (d) a link to a simple *self - assessment process* to determine a risk potential; (e) information and links to the Board 's website.'(Tanzanian, Internet Gaming regulations 2021)<br><br>'Art.60.- Gambling operators are required to take appropriate measures to protect players against gambling addiction and excessive commitment of wealth or income, in particular by: 1° *informing players*; 2° *the early identification of gamblers at risk*; 3° *player self-monitoring and limit setting*; 4° *adoption and application of exclusion measures*; 5° *training and development of their employees in responsible gambling*; 6° the collection of data necessary for the evaluation of the effectiveness of the measures taken ; 7° *promotion* of responsible gambling.' (Cote D'Ivoire, Law on Legal Regime for Games of Chance 2020)	Measures targeted at improving the evidence base and operators' accountability on gambling-related harms prevention. In some jurisdictions, these measures align closer with individual frame, while in others – with the system frame:<br><br>- conducting and/or funding research on gambling addiction;<br><br>- operators to report on the effectiveness of gambling harm prevention actions.	"§ 22 (Acceleration of inquiries and researches) *The State and any local public entity* shall as part of the measures against gambling addiction adopt necessary ones to— (a) accelerate inquiries and researches into the prevention of its onset, development and recurrence, diagnosis, remedy and other aspects of its issue; and (b) spread the results of the said inquiries and researches. § 23. (Field surveys) The Government shall every three years make surveys necessary to clarify the realities of the issue of gambling addiction and to publish its findings through the Internet and in any other appropriate manner" (Japan, Basic Law on Measures Against Gambling Addiction 2018)<br><br>"For the development, implementation and evaluation of the measures, *the casinos and the organizers of major games can work together with*: a. the competent law enforcement authorities; b. other casinos or other organizers of large games; c. *researchers*; i.e., addiction prevention centers; e. therapy facilities; f. Social Services." (Switzerland, Ordinance of the Swiss Confederation Casino Commission, 2018)<br><br>"*continuous documentation* of the measures taken for the purpose of drawing conclusions about *the effects of the games of chance offered on gaming behaviour and the emergence of gambling addiction*, as well as to assess the success of the measures taken to protect minors and players; 10. reporting to the gaming supervisory authorities or other competent permit authorities every two years based on the documentation as per subparagraph" (Germany, State Treaty on Gaming 2021)	Operators have responsibility to protect players from possible harms. They are required to partake in a range of supply-side and population-based prevention measures, including:<br>- mandatory player identification and mandatory limit setting;<br>- limiting availability and accessibility of gambling;<br>- restricting marketing (including advertising);<br>- restricting product design;<br>- allocating funding for prevention;<br>mandatory industry data sharing.	'§1 A licensee shall *ensure that social and health considerations are observed* in the gambling activities in order to protect players against excessive gambling and *help them to reduce their gambling* where there is a reason to do so (duty of care).' (Sweden, Gambling Act 2018)<br><br>"§ 6 Social concept (1) Organisers and brokers of public games of chance are *obliged to ensure the protection of minors and players*, to encourage players to engage in responsible gaming and to prevent gaming addiction. (2) For these purposes, the organisers and intermediaries of public gambling must develop and implement social concepts. The social concepts shall set forth the *measures that will be taken to prevent and remedy the socially harmful effects of gaming*. The social concepts must be *tailored to the different forms of games of chance*' (Germany, State Treaty on Gaming 2021)<br><br>"2. In order *to minimize the negative impact of gambling, gambling organizers are obliged* to adhere to the principles of responsible gambling, in particular:<br>1) *ensure the identification of the player (players) and the visitor (visitors)* in the manner specified by this Law in the gambling establishment and the identification of the player (players) when conducting activities on the Internet;<br>2) in cases and according to the procedure established by the Authorized Body, *prevent persons who have restricted access to gambling and persons with a pronounced gambling addiction from participating* in gambling games;<br>3) *refrain from providing players with any bonus payments, gifts and/or the provision of goods (services) in any form, use of other types of incentives*, the provision of which is directly or indirectly conditioned by the fact that the player loses in a relevant game." (Ukraine, Law on Organisation and Conduct of Gambling 2021)
	See Table 3 and Table S3 for specific measures, their frequency and distribution across cases					
